# Aer Receptors Influence the *Pseudomonas chlororaphis* PCL1606 Lifestyle

**DOI:** 10.3389/fmicb.2020.01560

**Published:** 2020-07-08

**Authors:** Eva Arrebola, Francisco M. Cazorla

**Affiliations:** ^1^Departamento de Microbiología, Faculta de Ciencias, Universidad de Málaga, Málaga, Spain; ^2^Instituto de Hortofruticultura Subtropical y Mediterránea “La Mayora” IHSM, UMA-CSIC, Málaga, Spain

**Keywords:** energy taxis, aerotaxis, bacteria, colonization, pseudomonas, avocado root

## Abstract

*Pseudomonas chlororaphis* PCL1606 (PcPCL1606) is a rhizobacterium isolated from avocado roots, which is a favorable niche for its development. This strain extensively interacts with plant roots and surrounding microbes and is considered a biocontrol rhizobacterium. Genome sequencing has shown the presence of thirty-one potential methyl-accepting chemotaxis proteins (MCPs). Among these MCPs, two candidates are putative functional aerotaxis receptors, encoded at locus PCL1606_41090 (*aer*1-1) and locus PLC1606_20530 (*aer*1-2), that are homologous to the Aer receptor of *Pseudomonas aeruginosa* strain PaO1. Single- and double-deletion mutants in one or both genes have led to motility deficiencies in oxygen-rich areas, particularly reduced swimming motility compared with that of wildtype PcPCL1606. No differences in swarming tests were detected, and less adhesion by the *aer* double mutant was observed. However, the single and double mutants on avocado plant roots showed delayed biocontrol ability. During the first days of the biocontrol experiment, the *aer*-defective mutants also showed delayed root colonization. The current research characterizes the presence of *aer* transductors on *P. chlororaphis*. Thus, the functions of the PCL1606_41090 and PCL1606_20530 loci, corresponding to genes *aer*1-1 and *aer*1-2, respectively, are elucidated.

## Introduction

Bacteria are ubiquitous in soils. They occupy and colonize niches with very high efficiency, and they can display a neutral, negative or beneficial interaction with plants (Alawiye and Babalola, 2019). The plant rhizosphere environment is often colonized by diverse microorganisms that modulate the physiology and morphology of the plant, enhancing plant growth through the promotion of hormones and serving as protectors against plant pathogens (Philippot et al., 2013). The plant rhizosphere attracts bacteria from the soil environment since plants can release as much as 40% of their photosynthetic fixed carbon through root exudates into the surrounding area (Hütsch et al., 2002; Kai et al., 2016). Thus, bacteria preferably colonize the root surface or rhizoplane and the adjunct soil zone or rhizosphere (Reinhold-Hurek et al., 2015). They take advantage of a constant flow of organic plant-based substrates, but in return, some of them promote plant growth by providing soluble inorganic nutrients and producing growth-promoting factors, and they can even provide protection against pathogens (Alawiye and Babalola, 2019).

Plant cultivars have great importance in the bacterial composition of the rhizosphere because of the different compounds they released through their roots. Various organic acids, such as aliphatic and aromatic acids, amides, carbohydrates (glucose and xylose), fructose, lactic malic, oxalic, pyruvic, succinic and amino acids, secreted from the roots play roles in chemotaxis and are called PGPR bioactive factors (Babalola, 2010). Root exudates and other root deposits cause physical and chemical changes to the soil rhizosphere, making it distinct from the bulk soil. The root exudates released are quickly assimilated by root-associated microbes and modified before being discharged into the rhizosphere soil by other microorganisms (Zhang et al., 2017). For this reason, the rhizosphere is home to an enormous number of diverse bacterial species, with the majority of culturable bacteria being gram negative (Babalola and Akindolire, 2011). Among these bacteria, rhizobacteria are characterized by aggressive colonization and subsequent establishment on plant roots (Alawiye and Babalola, 2019).

Motility and chemotaxis are essential interrelated characteristics for proper colonization of the rhizoplane. Motile bacteria that lack chemotaxis capacity are deficient at surface colonization sites; accordingly, the role of chemotaxis in surface colonization is clearly demonstrated (Tamar et al., 2015). Nutrients such as amino acids, sugars and organic acids from plant root exudates, terminal electron acceptors, light and temperature can affect tactic behavior, and they may all act as signals for different types of taxis. The sensing of specific cues is dependent on the presence of dedicated receptors, which are characterized by the specificity and sensitivity by which they detect signals (Alexandre, 2010). Chemoeffectors may physically bind to a receptor or to a specific periplasmic binding protein that interacts with a receptor (Alexandre, 2010). These cue transducers are known as methyl-accepting chemotaxis proteins (MCPs) and are relayed directly to the motility apparatus through chemotaxis signal transduction pathways (Bren and Eisenbach, 2000). When an extracellular compound binds to a membrane-bound MCP, it causes a phosphorylation signal cascade through CheA and CheY to alter the direction of flagellar rotation (Wadhams and Armitage, 2004), allowing the cell to direct the swimming direction, such as through a concentration gradient (Booth and Turner, 2016) to obtain optimized metabolic conditions or to avoid contact with toxic compounds; this behavior is traditionally known as bacterial chemotaxis.

On the other hand, effectors may also be detected indirectly by the receptor via their effects on intracellular energy levels. Their receptors act as energy sensors and are referred to as energy taxis receptors. Effectors of energy taxis include terminal electron acceptors, light, redox-active compounds and metabolizable substrates that act as donors for reduction that are equivalent to those in the electron transport system. Energy taxis differs from metabolism-independent taxis or chemotaxis only by the presence of dedicated energy taxis receptors (Alexandre, 2010). This ability has been attributed to the Aer receptors, which have been described and intensively studied in *Escherichia coli* (Rebbapragada et al., 1997; Edwards et al., 2006). Additional models of Aer transducers have been described in *Azospirillum brasilense*, namely, the Aer C receptor (Xie et al., 2010), and in *Pseudomonas aeruginosa*, namely, the Aer2 receptor (Hong et al., 2004). These kinds of transducers act when motile cells respond tactically to the direct effect(s) of stimuli on energy-generating processes or to effects resulting from the metabolism of a chemoeffector. Through energy taxis, cells do not navigate toward the greatest concentration(s) of effectors but seek positions where metabolic rates are optimized (Alexandre, 2010). Aerotaxis, taxis toward alternative electron acceptors, phototaxis, redox taxis and, in some bacterial species, chemotaxis toward metabolizable substrates have been described as forms of energy taxis (Taylor et al., 1999). One of the predicted advantages of energy taxis is that changes in tactic behavior are not dependent on the concentration of a specific effector, and the sensory signal that triggers the tactic response is the result of the integration of environmental conditions and the current metabolic activity of the cell (Alexandre, 2010).

There is published evidence confirming that energy taxis in plant-associated bacteria is metabolism dependent (Zhulin and Armitage, 1993; Armitage and Schmitt, 1997). In the plant-associated *Azospirillum* spp., aerotaxis and taxis toward rapidly used substrates (malate or succinate) are the dominant responses (Taylor et al., 1999). The carbon sources that support the fastest growth are also the best chemoattracts, which is typical of energy-dependent taxis. In addition to energy taxis toward metabolizable substrates in plant root exudates, redox taxis to substrates involved in the redox state of respiratory complexes, such as oxygen and other oxidable metabolites, may play major roles in plant-microbe interactions. The redox state of the rhizosphere is one of the most important parameters for maintaining this ecological system (Taylor et al., 1999).

*Pseudomonas chlororaphis* PCL1606 (PcPCL1606) is isolated from avocado roots and described as a very effective antagonist against *Rosellinia necatrix*, the pathogen causing white root rot of avocado (Cazorla et al., 2006). This bacterium produces the compound 2-hexyl, 5-propyl resorcinol (HPR), considered the main weapon against this pathogenic fungus (Calderón et al., 2013, 2014); this compound also plays an important role in the biofilm formation of PcPCL1606, directly influencing root colonization (Calderón et al., 2014, 2019). *Pseudomonas chlororaphis* strains typically produce phenazine antibiotics that are highly redox-active and would have a major role in the behavior of *aer* genes (Hernandez et al., 2004; Wang and Newman, 2008). However, this secondary metabolite is not produced by the model strain PcPCL1606 because it doesn’t harbor phenazine biosynthetic genes in its genome (Calderón et al., 2015). Recent studies have demonstrated that PcPCL1606 can detect compounds exuded by avocado roots and *R. necatrix*, which strongly attracts this rhizobacterium. Thus, PcPCL1606 has the capacity to detect compounds and move through soil specks toward a gradient of avocado exudates, as well as to *R. necatrix* exudates, which results in an efficient microbe-substrate interaction (Polonio et al., 2017). However, in this system, only one functional *che*A gene, corresponding to locus PCL1606_44390, according to its NCBI annotation, had been identified. Mutant PcPCL1606 strains constructed to be non-chemotactic (*che*A) and flagella-less (*flg*K) lacked the ability to respond in a chemotactic assay. Importantly, the *che*A mutant retained motility, in agreement with results previously described for the biocontrol of other PcPCL1606-*che*A mutants in *Pseudomonas* sp. (de Weert et al., 2002; Polonio et al., 2017). *In silico* analysis of the PcPCL1606 closed genome revealed sequences with high similarity to Aer-function-related genes, which are studied and characterized in the present study. Thus, the main goal has been verifying the energy taxis function and its involvement in the search and settlement of this antagonist in its optimal niche in the avocado root.

## Materials and Methods

### Bacterial Strains, Plasmids and Growth Conditions

Plasmids and bacterial strains are described in Table 1. *Escherichia coli* strains were grown on LB medium (Ausubel et al., 1995) at 37°C and supplemented with antibiotics according to the plasmid requirements. PcPCL1606 and defective mutants were also maintained on LB medium at 25°C, and the medium was supplemented with antibiotics as necessary (Table 1). The defined basal medium M9 (Rainey, 1999) supplemented with a carbon source (Nichols and Harwood, 2000) was used for the chemotaxis and energy taxis assays. The antibiotic concentrations used in this study were 50 μg/mL kanamycin (km) and 80 μg/mL gentamicin (Gm) for the PcPCL1606 defective mutants, 40 μg/mL for the *E. coli* strains, 80 μg/mL tetracycline (Tet) for PcPCL1606 and the defective deletion mutants in the pMP4655 GFP-expressing plasmid.

**TABLE 1:** Bacterial strains and plasmids used in this study.

Bacteria	Relevant characteristics 1	References
***Pseudomonas chlororaphis***		
PCL1606	Wildtype, biocontrol strain isolated from avocado rhizosphere, efficient root colonizer.	Cazorla et al., 2006
PCL1606::aer1-1	PCL1606 derivative insertional mutant in gene PCL1606_41090 [PCL1606_RS20050], Kmr	This study
PCL1606::aer1-2	PCL1606 derivative insertional mutant in gene PCL1606_20530 [PCL1606_RS10050], Kmr	This study
PCL1606Δaer1-1	PCL1606 derivative deletional mutant in locus PCL1606_41090 [PCL1606_RS20050].	This study
PCL1606Δaer1-2	PCL1606 derivative deletional mutant in locus PCL1606_20530 [PCL1606_RS10050].	This study
PCL1606Δaer	PCL1606 derivative deletional mutant in both loci PCL1606_20530 and PCL1606_41090	This study
COM-aer1-1	PCL1606::aer1-1 complemented with pLac4 Gmr and Kmr	This study
COM-aer1-2	PCL1606::aer1-2 complemented with pLac2 Gmr and Kmr	This study
PCL1606pBBR	PCL1606 complemented with empty cloning vector pBBR1MCS-5 Gmr	This study
PCL1606::aer1-1pBBR	PCL1606 derivative insertional mutant in locus PCL1606_41090 complemented with empty cloning vector pBBR1MCS-5 Gmr	This study
PCL1606::aer1-2pBBR	PCL1606 derivative insertional mutant in locus PCL1606_20530 complemented with empty cloning vector pBBR1MCS-5 Gmr	This study
PCL1606pLac2	PCL1606 complemented with pLac2 Gmr	This study
PCL1606pLac4	PCL1606 complemented with pLac4 Gmr	This study
PCL1606GFP	Wildtype PCL1606 expressing gfp protein by pMP4655 vector Tetr	This study
PCL1606Δaer1-1GFP	PCL1606 derivative deleted mutant in locus PCL1606_41090 expressing gfp protein by pMP4655 vector Tetr	This study
PCL1606Δaer1-2GFP	PCL1606 derivative deleted mutant in locus PCL1606_20530 expressing gfp protein by pMP4655 vector Tetr	This study
PCL1606ΔaerGFP	PCL1606 derivative deletional double mutant expressing gfp protein by pMP4655 vector Tetr	This study
***Escherichia coli***		
DH5αλpir	endA1 hsdR17 glnV44 (=supE44) thi-1 recA1 gyrA96 relA1 Φ80dlacΔ(LacZ)M15 Δ(LacZYA-argF) U169 zdg-232::Tn10 uidA::pir+	Platt et al., 2000
DH5α	dlacZ Delta M15 Delta(lacZYA-argF) U169 recA1 endA1 hsdR17(rK-mK+) supE44 thi-1 gyrA96 relA1	Taylor et al., 1993
**Plasmids**		
pBBR1MCS-5	Replicative cloning vector in Pseudomonas and Escherichia coli, Gmr	Kovach et al., 1995
pLac2	PCL1606_20530 [PCL1606_RS10050] cloned into pBBR1MCS-5, Gmr	This study
pLac4	PCL1606_41090 [PCL1606_RS20050] cloned into pBBR1MCS-5, Gmr	This study
pCR2.1	PCR products cloning vector lacZ, Kmr, Ampr	Invitrogen, CA, United States
pMut20530	A fragment of PCL1606_20530 [PCL1606_RS10050] sequence cloned into pCR2.1, Kmr for integrative mutation	This study
pMut41090	A fragment of PCL1606_41090 [PCL1606_RS20050] sequence cloned into pCR2.1, Kmr for integrative mutation	This study
pEMG	Integrative plasmid for deletion mutagenesis Kmr. Alternative plasmid’s name pJP5603-IScelv2.	Martínez-Garcia and de Lorenzo, 2012
pSW2	Plasmid with I-Scel gene for deletion mutagenesis, Gmr	Martínez-Garcia and de Lorenzo, 2012
pJ20530	pEMG carrying fragments of up and down of PCL1606_20530 [PCL1606_RS10050] for deletion mutagenesis Kmr	This study
pJ41090	pEMG carrying fragments of up and down of PCL1606_41090 [PCL1606_RS20050] for deletion mutagenesis Kmr	This study
pMP4655	plasmid to express autofluorescent protein GFP in gram-negative bacteria, Tetr	Bloemberg et al., 2000

*1. In square brackets [..] alternative name of locus of PCL1606_20530 and PCL1606_41090 found in National Center for Biotechnology Information (NCBI). Ampicillin (Amp), Gentamicin (Gm), Kanamycin (Km), Tetracycline (Tet), green fluorescent protein (gfp).*

### *In silico* Sequence Analysis and Phylogenetic Analysis

The PcPCL1606 closed genome was deposited in the National Center for Biotechnology Information (NCBI) database with accession number NZ_CP011110.1. A manual search of the GenBank genome annotation for PcPCL1606 with putative MCP and PAS protein sequences was performed. The EMBL-EBI database was used to search for potential functions of the MCPs with the HMMER tool: biosequence analysis using profile hidden Markov models^1^. PcPCL1606 loci sequences and other bacterial sequences used in the present study were obtained from the NCBI database. Similarly, a nucleotide BLAST analysis was used for the sequence analysis, and the conserved domain database was used for the domain structure analysis. Queries of promoters and terminators of putative *aer* genes in PcPCL1606 were performed on the Softberry website^2^ using BPROM and FindTerm software. The location of the transmembrane sequence in the Aer proteins of *P. chlororaphis* strains PCL1606, B25 and ATCC13985, *P. aeruginosa* PaO1 and *E. coli* K-12 were determined by the DAS-TMfilter server^3^ (Cserzo et al., 2004). The European Bioinformatics Institute^4^ website was accessed for Clustal Omega (1.2.4) analysis, which was used for multiple amino acid sequence alignment.

Molecular phylogenetic analysis by the maximum likelihood method was used to generate a phylogenetic tree. The evolutionary history was inferred by using the maximum likelihood method based on the JTT matrix-based model (Jones et al., 1992). The bootstrap consensus tree inferred from 100 replicates (Felsenstein, 1985) was taken to represent the evolutionary history of the taxa analyzed (Felsenstein, 1985). Branches corresponding to the partitions reproduced in less than 50% of the bootstrap replicates were collapsed. The percentage of replicate trees in which the associated taxa clustered together in the bootstrap test (100 replicates) is shown next to the branches (Felsenstein, 1985). Initial tree(s) for the heuristic search were obtained automatically by applying neighbor-joining and BIONJ algorithms to a matrix of pairwise distances estimated using the JTT model and then selecting the topology with the superior log likelihood value. The analysis involved 57 amino acid sequences. All positions containing gaps and missing data were eliminated. There was a total of 413 positions in the final data set. Evolutionary analyses were conducted in MEGA7 (Kumar et al., 2016). The bacterial strains and sequence and amino acid accession numbers are listed in Supplementary Table S1.

GC percentage and DNA length were obtained by the DNA/RNA GC content calculator website^5^.

### Strain Manipulation and Molecular Assays

Insertion mutagenesis inactivated PcPCL1606 was used to test energy taxis level; specifically, in a disruption vector was inserted into putative *aer* genes located on the PcPCL1606 chromosome via single-crossover homologous recombination (Arrebola et al., 2012). The cloning of vector pCR2.1 (Invitrogen Life Technologies United States) and plasmid purification were performed using standard procedures. The plasmids were transformed into wildtype PcPCL1606 by electroporation (Choi et al., 2006).

On the other hand, deletion mutagenesis was used to obtain clean mutants with the putative *aer* genes completely removed (Martínez-Garcia and de Lorenzo, 2012). The pEMG plasmid, containing fragments up- and downstream from the putative *aer* gene, was used for deletion mutagenesis, according to the protocol described Martínez-Garcia and de Lorenzo (2012), and the complementation vectors pLac2 and pLac4 (Table 1) were obtained using NEBuilder HiFi DNA Assembly Master Mix (New England Biolabs, GmbH., Inc.).

The correct insertions of the disruption vectors and clean *aer* gene deletion were verified by PCR. The bacterial growth curves were obtained in LB and M9 culture media to confirm similarities among the constructed defective mutants and the wildtype PcPCL1606 (data not shown).

For complementation, the replicative vector pBBR1MCS-5 was used to clone the complete CDS of the putative *aer* genes corresponding to locus PCL1606_20530 and locus PCL1606_41090. All primers involved in mutagenesis and complementation are listed in Supplementary Table S2.

### Taxis to Atmospheric Air

For the air trap assay, bacteria were grown at 25°C in LB agar medium supplemented with antibiotics, as necessary, for 20 to 24 h. These bacteria were resuspended in motility buffer (Parkinson and Houts, 1982; Bibikov et al., 1997), with the optical density at 600 nm of 1 (OD_600_ at (1). A total of 150 μL of bacteria culture was injected by syringe into a tube with LB dense broth (with 0.1% bacteriological agar). The tubes with the LB dense broth included a Pasteur pipette that protruded 2 or 3 cm from the LB medium and closed with a cap and previously sterilized by autoclave for 15 min at 121°C. The bacterial sample was deposited into the LB dense broth but outside of the Pasteur pipette. After inoculation, 2 mL of paraffin oil was added to the LB dense broth but was prevented from falling into the Pasteur pipette. In this way, air exchange was avoided in the broth but not in the Pasteur pipette, thus forming an air trap. In this assay, we analyzed the attraction of the air by the bacteria. After 24 h of incubation at 25°C, 100 μL of the air-medium contact surface in the Pasteur pipette was recovered, and the bacterial counts were analyzed. Three independent experiments with five replicates of each were performed. Statistical data analysis was based on the Kruskal-Wallis test (one-way of variance on ranks) (SigmaPlot 12.0) was performed.

To improve microscopic visualization of bacterial displacement, we conducted a more detailed study on PcPCL1606 and the defective mutant behavior in the presence of atmospheric air, optical microscope observations were performed. Bacteria were grown under the same conditions as those used in the air trap assay, with the OD_600_ at 0.5. A 3 μL drop of bacteria culture was deposited on a coverslip, and it was placed on an excavated slide. In this way, the drop was surrounded by atmospheric air without pressure that could modify the bacterial behavior. The bacterial air taxis was observed by optical microscopy at 1000 × magnification. Photographs of the same field were taken at 5, 10, 15, and 20 min to monitor the bacterial behavior. The light was turned off between photographs to prevent its effect on the sample. The number of bacteria located in the drop edge in every sample time was counted, and the mean was obtained from three photographs from different observation fields. Statistical data analysis using one-way analysis of variance with Shapiro-Wilk normality tests followed by all pairwise multiple comparisons (Tukey test) *p* < 0.05 (SigmaPlot 12.0) was performed.

### Analysis of Alternative Electron Acceptors

A nitrate reduction test in aerobiosis and anaerobiosis systems was conducted to determine the capacity of wildtype PcPCL1606 and the defective mutants to reduce nitrate to nitrite or to gas nitrogen, thereby determining the likelihood that the strains use nitrate as a final electron acceptor in cases of decreased oxygen concentration. Furthermore, a chemotaxis analysis (Polonio et al., 2017) using nitrate at 2, 0.2, and 0.02% and another electron source with DMSO as the attractant was performed in both aerobiosis and anaerobiosis environments. For these experiments, soft agar plates with M9 medium supplemented with succinic acid, amended with 0.2% bacteriological agar, were used. The plates were inoculated with ten microliters of bacterial suspension with OD_600_ at 0.3 and incubated in an anaerobiosis system (GasPak^TM^ EZ anaerobe container system, Fisher, United States) or a non-hermetic incubator for anaerobiosis or aerobiosis tests, respectively. The cultures were incubated at 25°C for 3 days for the aerobiosis test and 5 days for the anaerobiosis test.

### Motility Analysis in Soft Agar

The ability of PcPCL1606 to assimilate different sugars was analyzed. The Hugh Leifson sugar metabolism test (Hugh and Leifson, 1953) was used with glucose, arabinose, glycerol, maltose, mannitol, succinate, sorbitol, fructose, galactose, raffinose, sucrose, trehalose, xylose, dulcitol, and lactose. The sugars for which negative results were obtained were discarded. Different concentrations were tested with the selected sugars to determine which presented the largest motility differences. The concentrations analyzed were 10, 8, 6, 4, and 2 mM. The motility results for the different concentrations were very similar for each sugar; therefore, 2 mM was chosen for the soft agar test for the sugars and organic acids, such as glutamic acid, succinic acid and malic acid. The data were obtained after three independent experiments with three replicates.

To test the bacterial motility, the strains were grown in LB agar medium or LB agar medium with antibiotics at 25°C for 20–24 h, and the bacterial strains were stab inoculated with sterile toothpicks in soft M9 agar with 0.3% bacteriological agar. These soft M9 agar plates were supplemented with 2 mM arabinose, fructose, galactose, glycerol, glucose, sucrose, xylose, glutamic acid, malic acid, and succinic acid (Bibikov et al., 1997; Greer-Phillips et al., 2003). All stock solutions were previously adjusted to a neutral pH. The motility was measured after 24 h of incubation at 25°C according to the diameter of the motility front for three independent experiments with three replicates. Motility values from the defective mutants and complement strains were normalized to those of the wildtype strain. Data were analyzed by Kruskal-Wallis test (one-way analysis of variance on ranks) (SigmaPlot 12.0).

Swarming motility was analyzed to observe possible motility differences between wildtype PcPCL1606 and the defective deletion mutants to determine their potential colonization abilities. For this determination, plates with 15 mL of TPG medium (Calderón et al., 2014) with 0.5% bacteriological agar were inoculated with 3 μL of bacterial suspension, with the adjusted OD_600_ at 0.8. The inoculated plates were incubated at 25°C for 48 h, and three independent experiments with three replicates each were performed for statistical analysis. Further, a chemotaxis test (Polonio et al., 2017) with each carbon source used was conducted to determine whether the chemotactic ability was affected in the PcPCL1606 defective mutants. The results were analyzed by Kruskal-Wallis test (one-way analysis of variance on ranks) (SigmaPlot 12.0).

### Adhesion Assay

Bacterial adhesion on glass surfaces was analyzed using a static microcosm assay (Pliego et al., 2008) in combination with crystal violet dye (Peeters et al., 2008). Briefly, static microcosms were studied using sterile staining bottles containing 15 mL of LB broth and five sterile object slides (25 mm × 15 mm × 1.5 mm) placed inside separately. LB cultures that had been shaken overnight were used to inoculate the microcosms to densities of approximately 10^7^ cfu/mL (OD_600_ at 0.1). Microcosms were incubated at 25°C for 8 h, and samples were taken every hour, and the final samples were taken at 24 and 28 h.

The crystal violet assay (Peeters et al., 2008) was used to quantify biofilm formation; that is, the stained bacteria attached to the glass surface. Every hour, a slide from each strain culture was taken and immersed for 15 min in 99% methanol for fixation. Then, the object slides were air-dried and immersed in 1% crystal violet for 20 min. Next, the excess crystal violet was removed by washing the slides under running tap water. Finally, the bound crystal violet was released from the object slides with added bacteria stained by dipping in 15 mL of 33% acetic acid. The absorbance of a 1 mL sample of the crystal violet extraction was measured at 590 nm. Five independent experiments with two replicates of each were performed for the statistical analysis. One-way analysis of variance with Shapiro-Wilk normality test followed by all pairwise multiple comparisons (Tukey test) *p* < 0.05 (SigmaPlot 12.0) was performed.

### Bacterial Interaction on the Avocado Rhizosphere

Biocontrol assays against white root rot were carried out in the avocado-*Rosellinia necatrix* system (Cazorla et al., 2006). Six-month-old commercial avocado plants (cv. Walter Hole) sprouted from seeds were obtained from Brokaw nurseries (Brokaw España S.L., Velez-Málaga, Spain). The plants were carefully removed from the pots to avoid breaking the roots, and they were washed in tap water; then, the roots were disinfected by immersion in 1% commercial bleach (0.4 g/L of sodium hypochloride) for 20 min and washed in sterile distilled water for another 20 min (Cazorla et al., 2006). Disinfected avocado roots were bacterized following the method published by Lugtenberg et al. (1994) and slightly modified by Calderón et al. (2013). Three independent groups of five plants each underwent the following inoculations: wildtype PcPCL1606 as a positive control, only the high virulence and wide range pathogen *R. necatrix* as a negative control, single-deletion mutants PCL1606Δaer1-1 and PCL1606Δaer1-2, double-deletion mutant PCL1606Δaer, and fifteen plants without any treatment as method controls. The treated avocado plants were maintained in a monitored greenhouse at 25°C and 75% RH. The number of diseased seedlings was determined 4 weeks after bacterization, recording the evolution of the disease every few days. Aerial symptoms were recorded on a scale of 0 to 3, and the disease index (DI) was then calculated using the previously described method by Cazorla et al. (2006). Independent plant experiments were performed twice, and data analysis was performed by *T*-test with α = 0.05 and 4 degrees of freedom (SigmaPlot 12.0).

For short-term colonization assays and observation by confocal laser-scanning microscopy (CLSM) Leica SP5, Leica Microsystems, Bensheim, Germany), 1-month avocado seedlings were used. The plants were disinfected as described previously, and the avocado cotyledons were carefully removed. These disinfected plants were then introduced into gnotobiotic tubes, as similarly, described in a previous work (Simons et al., 1996). Gnotobiotic avocados were inoculated with 1 mL of each strain adjusted to 10^8^ cfu/mL (OD_600_ at 0.8) in sterile distilled water and deposited on the neck of the plant. Five independent gnotobiotic plants of each strain marked with GFP, wildtype PcPCL1606GFP, single-deletion mutants PCL1606Δaer1-1GFP and PCL1606Δaer1-2GFP, and double-deletion mutant PCL1606ΔaerGFP, were obtained (Table 1). The gnotobiotic plants were placed into a growth chamber (25°C, cycles of 16 h of light and 8 h dark). Twenty-four hours after inoculation, one plant of each strain was selected, and 3 and 5 days after inoculation, two plants for each strain were selected; these selected plans were subjected to CLSM at 630× magnification. Five root tips per plant were observed. Three to five photographs were obtained for each strain at every sampling time.

Colonization and persistence over a longer period were measured by bacterial count. For these assays, 6-month avocado plants were disinfected as described above and placed in pots with sterile vermiculite. Then, the plants were acclimatized for 2 days in a growth chamber (at 25°C and om cycles of 16 h light–8 h dark). Next, wildtype PcPCL1606 and the defective single- and double-deletion mutants were inoculated as previously described for the colonization assay. Nitrofurantoin (50 μg/mL) and cycloheximide (100 μg/mL), which confer natural resistance, were used to avoid bacterial and fungal contamination.

Data from the short and long colonization and persistence assays were analyzed for significance using one-way analysis of variance with the Shapiro-Wilk normality test followed by all pairwise multiple comparisons (Tukey test), *p* < 0.05 (SigmaPlot 12.0).

## Results

### Molecular Analysis of Two Putative *aer* Genes in PcPCL1606

*In silico* analysis of the PcPCL1606 genome showed the presence of 31 proteins considered putative methyl-accepting chemotaxis proteins (MCPs), two of which have putative functions as aerotaxis receptors (Table 2), according to the database. These two loci, PCL1606_20530 (also known as PCL1606_RS10050) and PCL1606_41090 (PCL1606_RS20050), belong to the PAS-Box family of proteins that were found in the PcPCL1606 genome in a second *in silico* analysis. Therefore, PCL1606_20530 and PCL1606_41090 are the only two loci with shared MCPs and PAS-Box protein sequences in the PcPCL1606 genome.

**TABLE 2:** *In silico* detection of putative methyl-accepting chemotaxis protein located in *Pseudomonas chlororaphis* PCL1606 genome.

Number	Locus	Protein_id	Protein size (aa)	Putative function	Specie compared and strain	*E*-value
1	PCL1606_01730	WP_045880560.1	658	Calcium chemotaxis sensor transducer	*Pseudomonas fluorescens* ATCC BAA-477, NRRL-B-23932, Pf5	0.0e+00
2	PCL1606_02700	WP_045880659.1	535	Aspartate chemoreceptor protein	*Pseudomonas fluorescens* ATCC BAA-477, NRRL-B-23932, Pf5	4.6e-256
3	PCL1606_03870	WP_044466183.1	689	Type IV pili methyl-accepting chemotaxis transducer	*Pseudomonas fluorescens* ATCC BAA-477, NRRL-B-23932, Pf5	0.0e+00
4	PCL1606_10550	WP_044465078.1	438	Methyl-accepting chemotaxis sensory transducer with Pas/Pac sensor	*Pseudomonas* sp. OK602	7.5e-233
5	PCL1606_10560	WP_045881254.1	438	Methyl-accepting chemotaxis sensory transducer with Pas/Pac sensor	*Pseudomonas mucidolens*^2^	4.5e-184
6	PCL1606_11440	WP_045881344.1	713	Chemotaxis protein	*Pseudomonas fluorescens* ATCC BAA-477, NRRL-B-23932, Pf5	0.0e+00
7	PCL1606_14760	WP_044464692.1	629	Chemotaxis protein	*Pseudomonas fluorescens* HK44	0.0e+00
8	PCL1606_15490	WP_044464634.1	712	Methyl-accepting chemotaxis protein	*Pseudomonas fluorescens* ATCC BAA-477, NRRL-B-23932, Pf5	0.0e+00
9	PCL1606_16310	WP_045881754.1	541	Methyl-accepting chemotaxis protein	*Pseudomonas fluorescens* ATCC BAA-477, NRRL-B-23932, Pf5	1.4e-288
10	PCL1606_16400	WP_045881767.1	439	Methyl-accepting chemotaxis sensory transducer with Pas/Pac sensor	*Pseudomonas asplenii*^2^	1.9e-206
11	PCL1606_18870	WP_008079890.1	493	Chemotaxis protein	*Pseudomonas fluorescens* HK44	8.9e-275
12^1^	**PCL1606_20530**	WP_044464188.1	521	Putative aerotaxis receptor	*Pseudomonas fluorescens* ATCC BAA-477, NRRL-B-23932, Pf5	1.7e-241
13	PCL1606_21870	WP_045882190.1	497	Methyl-accepting Chemotaxis protein	*Pseudomonas fluorescens* F113	1.3e-179
14	PCL1606_22470	WP_045882253.1	559	Chemotaxis protein	*Pseudomonas fluorescens* ATCC BAA-477, NRRL-B-23932, Pf5	0.0e+00
15	PCL1606_27190	WP_044463443.1	536	Histidine kinase, chemotaxis sensory transducer	*Pseudomonas* sp. STFLB209	2.3e-220
16	PCL1606_31390	WP_045883217.1	538	Chemotaxis protein	*Pseudomonas fluorescens* F113	1.5e-288
17	PCL1606_36090	WP_009043388.1	525	Chemotaxis protein	*Pseudomonas fluorescens* ATCC BAA-477, NRRL-B-23932, Pf5	1.0e-259
18	PCL1606_37880	WP_045884091.1	542	Chemotaxis protein	*Pseudomonas fluorescens* ATCC BAA-477, NRRL-B-23932, Pf5	4.1e-264
19^1^	**PCL1606_41090**	WP_044462163.1	521	Aerotaxis receptor Aer	*Pseudomonas fluorescens* ATCC BAA-477, NRRL-B-23932, Pf5	0.0e+00
20	PCL1606_43360	WP_045884778.1	654	Chemotaxis protein	*Pseudomonas fluorescens* HK44	0.0e+00
21	PCL1606_43600	WP_044461944.1	541	Chemotaxis protein	*Pseudomonas* sp. MF4836	3.6e-283
22	PCL1606_49730	WP_044461408.1	654	Serine chemoreceptor protein	*Pseudomonas* sp. FeS53a	9.2e-176
23	PCL1606_52530	WP_045885705.1	676	Methyl-accepting chemotaxis protein	*Pseudomonas* sp. MF4836	0.0e+00
24	PCL1606_53160	WP_045885777.1	639	Chemotaxis protein	*Pseudomonas* sp. MF4836	0.0e+00
25	PCL1606_53840	WP_044461000.1	648	Chemotaxis protein	*Pseudomonas fluorescens* HK44	0.0e+00
26	PCL1606_54190	WP_045885918.1	654	Methyl-accepting chemotaxis protein CtpL	*Pseudomonas fluorescens* ATCC BAA-477, NRRL-B-23932, Pf5	0.0e+00
27	PCL1606_55170	WP_045886051.1	606	Chemotaxis protein	*Pseudomonas fluorescens* ATCC BAA-477, NRRL-B-23932, Pf5	0.0e+00
28	PCL1606_55940	WP_044460813.1	541	Methyl-accepting chemotaxis protein NahY	*Pseudomonas fluorescens* ATCC BAA-477, NRRL-B-23932, Pf5	4.3e-243
29	PCL1606_56670	WP_045886252.1	638	Chemotaxis protein	*Pseudomonas fluorescens* ATCC BAA-477, NRRL-B-23932, Pf5	0.0e+00
30	PCL1606_56920	WP_045886282.1	626	Chemotactic transducer PctC	*Pseudomonas fluorescens* ATCC BAA-477, NRRL-B-23932, Pf5	0.0e+00
31	PCL1606_59520	WP_044460496.1	626	Chemotactic transducer PctA	*Pseudomonas fluorescens* ATCC BAA-477, NRRL-B-23932, Pf5	0.0e+00

*Putative function obtained by amino acid sequences and domains comparison by HMMER-Biosequence analysis using profile hidden Markov Models (PHMMER tool: https://www.ebi.ac.uk/Tools/hmmer/search/phmmer). 1.- In gray putative aerotaxis receptors. 2.- Strain not shown by data base. In bold letters loci selected for analysis in the present study.*

A nucleotide comparison of both loci, PCL1606_20530 and PCL1606_41090, with the database indicated that they had high similarity with Aer proteins, which are described as having functions as aerotaxis transducers. Specifically, PCL1606_20530 showed 77% identity and 94% coverage, while PCL1606_41090 showed 80% identity and 100% coverage with the aerotaxis receptor *aer* of *Pseudomonas aeruginosa* strain PaO1 (Hong et al., 2004). These two putative genes of PcPCL1606 have 80% of nucleotide and 74% of amino acid similarity. With respect to the levels of similarity of *P. aeruginosa* PaO1 loci, we refer to locus PCL1606_41090 as gene *aer*1-1 and PCL1606_20530 as gene *aer*1-2 in this study.

With Softberry software, an *in silico* study of the features of each locus in PcPCL1606 showed that *aer*1-1 (PCL1606_41090) is encoded in coding sequence positions from 4507037 to 4508602, consisting of 1566 bp (521 aa). It presented with only one putative promoter, 113 bp before the start codon, with a -10 box at position 98 (-TTCTCAAAT-) and a -35 box at position 74 (-TTCAAG-), without known sequences at the transcriptional binding site. A terminator search revealed only one possible position, at 1572 bp and 37 nucleotides in length, according to FindTerm software of Softberry Inc. On the other hand, *aer*1-2 (PCL1606_20530) is encoded in a complement sequence, position c2148064-2149629, and it also has 1566 bp (521 aa), identical to *aer*1-1. It presented with only one putative promoter 160 bp before the start codon with a -10 box at position 145 (-GACTAAGAT-) and a -35 box at position 122 (-ATGCAA-). Further, oligonucleotides were located based on known transcriptional factor binding sites: marR (-CAACTAAT-) at position 110, *gcv*A (-AACTAATT-) at 111, *pho*B (-TTTTCATA-) at 132 and *pho*B (-TCATAAAA-) at 135, according to the BPROM analysis. A search for a Rho-independent bacterial terminator sequence showed two possible positions: one in the open reading frame (ORF) starting at nucleotide 1272 and 40 bases in length, and another putative terminator outside the ORF starting at nucleotide 1597 and 42 bases in length.

**FIGURE 1:**
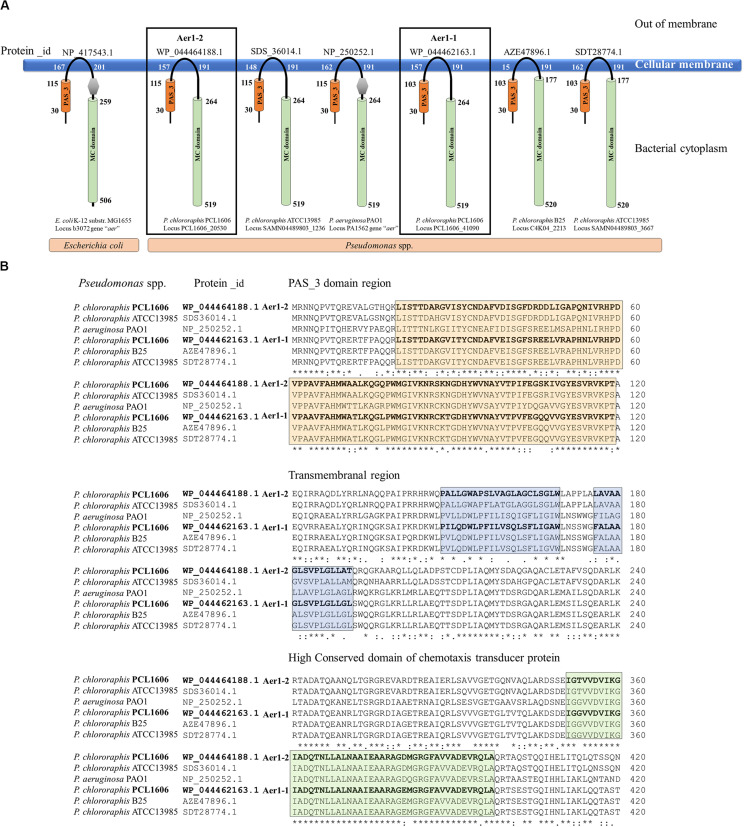
*In silico* analysis of the two loci located in the PCL1606 genome (PCL1606_20530 and PCL1606_41090) related to aerotaxis. **(A)** Model of the spatial structure and distribution of typical domains in Aer receptor proteins. Aer receptors of *E. coli* K-12 and *P. aeruginosa* PaO1 have been used as references. The PAS domain is shown as an orange cylinder, and the first and last amino acids in the domain are shown. The methyl-accepting chemotaxis-like domain (MC domain) is the green cylinder, and the first and last amino acids are present. The DUF454 domain is in a gray hexagon, and the transmembrane zone is also located by the first and last amino acids. The protein_id of each protein is on the top, and bacterial species and locus number are below each scheme. **(B)** Amino acid sequence comparison performed of six Aer receptors by Clustal-Omega is shown in section A. Protein_id WP_044464188.1 corresponding to *Pseudomonas chlororaphis* strain PCL1606 locus PCL1606_20530 (Aer1-2); protein_id SDS36014.1 corresponding to *P. chlororaphis* strain ATCC13985 locus SAMN04489803_1236; protein_id NP_250252.1 corresponding to *Pseudomonas aeruginosa* strain PaO1 locus PA1561; protein_id WP_044462163.1 corresponding to *P. chlororaphis* strain PCL1606 locus PCL1606_41090 (Aer1-1); protein_id AZE47896.1 corresponding to *P. chlororaphis* strain B25 locus C4K04_2213 and protein_id SDT28774.1 corresponding to *P. chlororaphis* strain ATCC13985 locus SAMN04489803_3667. The orange box frames the amino acids belonging to the PAS domain, the blue box frames the amino acids belonging to the transmembrane region, and the green box frames the amino acid sequences that are highly conserved in the chemotaxis domain.

**FIGURE 2:**
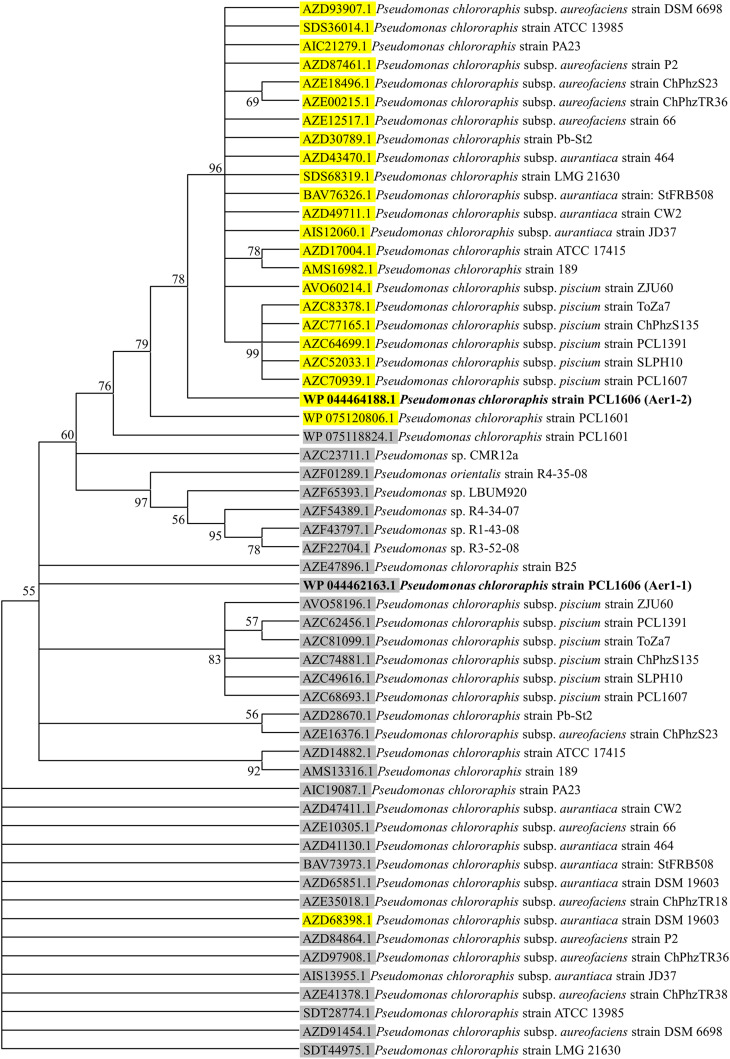
Consensus tree of phylogenetic relationships of Aer receptor proteins found in the genomes of the *Pseudomonas* sp. and *Pseudomonas chlororaphis* strains, including subspecies *aureofaciens*, *aurantiaca* and *piscium*. The protein_id information of the proteins with greater similarity to those corresponding to locus PCL1606_20530 are highlighted in yellow, and the protein_id information of the proteins with greater similarity to those corresponding to locus PCL1606_41090 are highlighted in gray. Protein_id WP044464188.1 corresponding to *Pseudomonas chlororaphis* PCL1606 (PcPCL1606) locus PCL1606_20530 (Aer1-2) is shown in bold, as is protein_id WP044462163.1 corresponding to PcPCL1606 locus PCL1606_41090 (Aer1-1). For information about the sequences used in the consensus phylogenetic tree, see Supplementary Table S1.

A deeper analysis revealed that the two genes of PCL1606 showed the same number and type of domains as the *aer* genes of *E. coli* K-12, *P. aeruginosa* PaO1, and other similar proteins of *P. chlororaphis* strains (Figure 1A), all of which have transmembrane regions between the PAS domain and methylation region. The sequence belonging to each domain of *Pseudomonas* sp., shown in Figure 1A, was located using a multiple amino acid comparison (Figure 1B), with the *aer* amino acid sequence of *P. aeruginosa* PaO1 used as a reference for determining domain locations (Nichols and Harwood, 2000). The most variable sequence was located in the transmembrane region, where it is possible to distinguish two types: one group characterized by protein WP_044464188.1 of strain PCL1606 and protein SDS36014.1 of strain ATCC13985 and the other group characterized by protein WP_044462163.1 of PCL1606, AZE47896.1 of strain B25 and SDT28774.1 of ATCC13985. The high similarity observed prompted the search for similar loci in *Pseudomonas chlororaphis* subspecies using a phylogenetic analysis (Figure 2). Thirty-three strains were assessed, five strains of *Pseudomonas* sp., nine strains of *P. chlororaphis*, five strains of subspecies *aurantiaca*, six of subsp. *piscium*, seven of subsp. *aureofaciens* and one strain of *Pseudomonas orientalis* (Supplementary Table S1). Except for seven strains, all had two loci similar to strain PcPCL1606. Both loci were located separately in the phylogenetic tree, resulting in two groups, one with higher similarity to *aer*1-1 and the another with higher similarity to *aer*1-2. The *aer*1-1 group was slightly broader because it included the loci found in the seven strains where only one locus had been found. The only exception is the protein AZD68398.1 of *P. chlororaphis* subsp. *aurantiaca* strain DSM19603, for which the phylogenetic analysis placed it in the locus PCL1606_41090 group (*aer*1-1 like protein), although it had greater similarity with the locus PCL1606_20530 (*aer*1-2 like protein) according to the protein comparison. In fact, the results obtained by BLAST analysis indicated that it had 100% coverage and 74.3% identity with *aer*1-1 and 100% coverage and 95% identity with aer1-2 at the amino acid level and 96% coverage and 80.3% identity with *aer*1-1 and 100% coverage and 91.89% identity with *aer*1-2 at the nucleotide level.

The calculation of the GC percentage in all genes used to generate the phylogenetic tree (59 coding sequences or CDS, corresponding to 33 CDS in the *aer*1-1-like protein group, and 26 CDS in the *aer*1-2-like protein group) revealed that the CDS in the *aer*1-1-like protein group had a GC content that ranged between 63.09 and 65.32%, while the CDS in the *aer*1-2-like protein group had a GC content that ranged between 66.4 and 68.3%, however, all 59 genes were 1566 nucleotides (521 aa) in length without exception. The homogeneity shown by the *Pseudomonas* sp. strains led to the further study of the genetic background of both genes. Genes located upstream from *aer*1-1 in the PcPCL1606 strain encoded products such as aconitate hydratase and CAAX amino terminal protease, and the genes downstream encoded alpha/beta hydrolase and cytochrome C oxidase. This genetic background was the same for the great majority (97%) of homologous genes analyzed in other *Pseudomonas* sp. and *P. chlororaphis* strains.

Regarding the *aer*1-2 genetic background, genes with the following functions were identified: hypothetical protein (PLC1606_20510) and peptide chain release factor-like protein (PCL1606_20520) for upstream genes. The products of the downstream genes were hypothetical proteins for PCL1606_20540 and the HAD family of hydrolases for PCL1606_20550. The genetic background for homologous genes in other *Pseudomonas* strains used to generate the phylogenetic tree revealed that the products of the genes near putative Aer transductors varied between β-phosphoglucomutase and haloacid dehalogenase; periplasmic lysozyme inhibitor and a hypothetical protein; and aldehyde dehydrogenase and a hemerythrin-domain protein. The most frequent order of arrangement in *P. chlororaphis* subspecies is as follows: β-phosphoglucomutase, hypothetical protein, aerotaxis receptor Aer, periplasmic lysozyme inhibitor and aldehyde dehydrogenase (Supplementary Figure S1).

### Taxis of PcPCL1606 to Air

**FIGURE 3:**
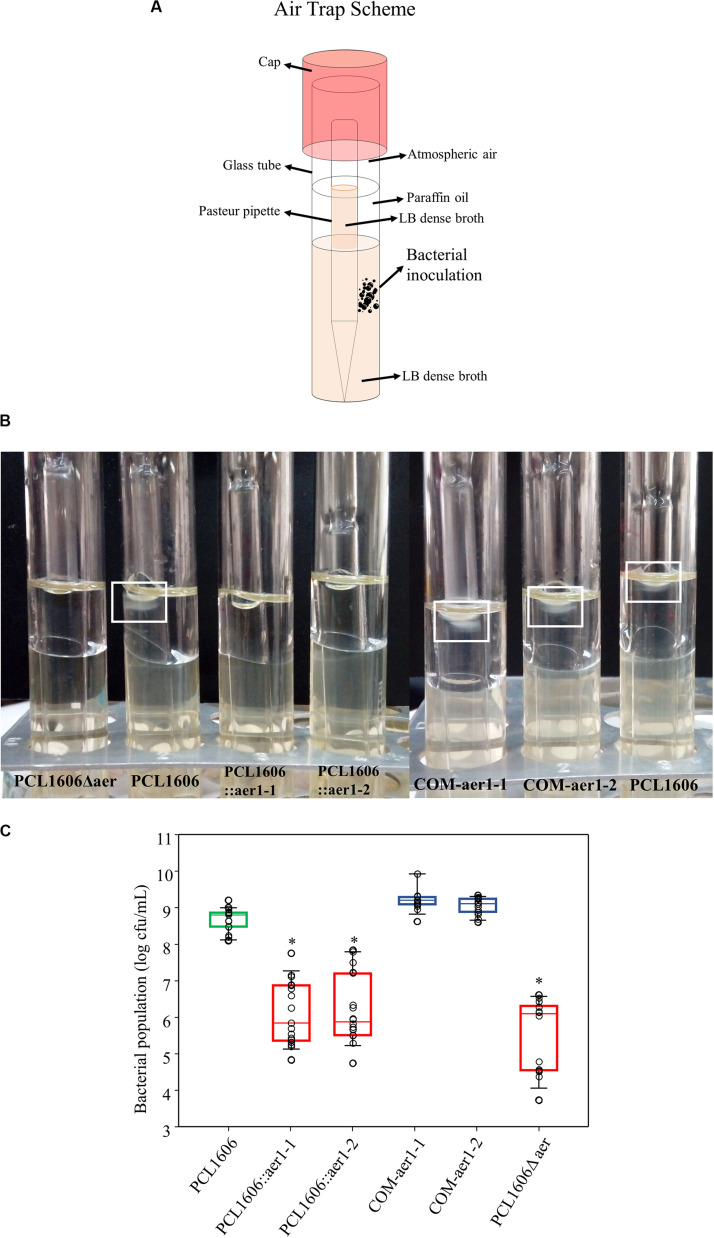
Air trap experiments of wildtype *Pseudomonas chlororaphis* PCL1606 (PcPCL1606), defective insertion mutants PCL1606::aer1-1 and PCL1606::aer1-2 and their defective complemented strains COM-aer1-1 and COM-aer1-2, and double-deletion mutant PCL1606Δaer. **(A)** Scheme of the air trap tubes designed for the experiment. Briefly, the air trap assay consisted of tubes with LB dense broth with sterile Pasteur pipette inserted in it. Paraffin oil was added to the broth but not the Pasteur pipette, which served as the air trap. **(B)** Photography showing bacterial behavior in the air trap system. When air is detected and acts as an attractant, the bacteria move and grow close to the medium surface inside the Pasteur pipette, which is free of paraffin oil. A white square marks wildtype PcPCL1606 growth after incubation. **(C)** Bacterial counts, expressed in decimal logarithm per milliliter, present in LB medium surface after 24 h of incubation at 25°C. Strains are represented as follows: wildtype PcPCL1606 in green; defective insertional and deletional mutants, singles and double, in red; and complemented mutants in blue. Points with asterisks indicate significant differences from wildtype PcPCL1606. Statistical analysis by Kruskal-Wallis test (one-way of variance on ranks) (SigmaPlot 12.0).

Once *aer*1-1 and *aer*1-2 were determined to be possible energy taxis or aerotaxis receptor genes, experiments using defective single- and double-deletion mutants were performed to verify the direct relationship among taxis, atmospheric air and these two genes. Using air trap experiments (Figure 3A), a higher bacterial concentration was observed in the medium-air contact surface in the Pasteur pipette when both genes were activated or a mutation was complemented. However, the absence of the activity of either gene or both genes resulted in bacterial growth two orders of magnitude lower in the same zone (Figures 3B,C).

**FIGURE 4:**
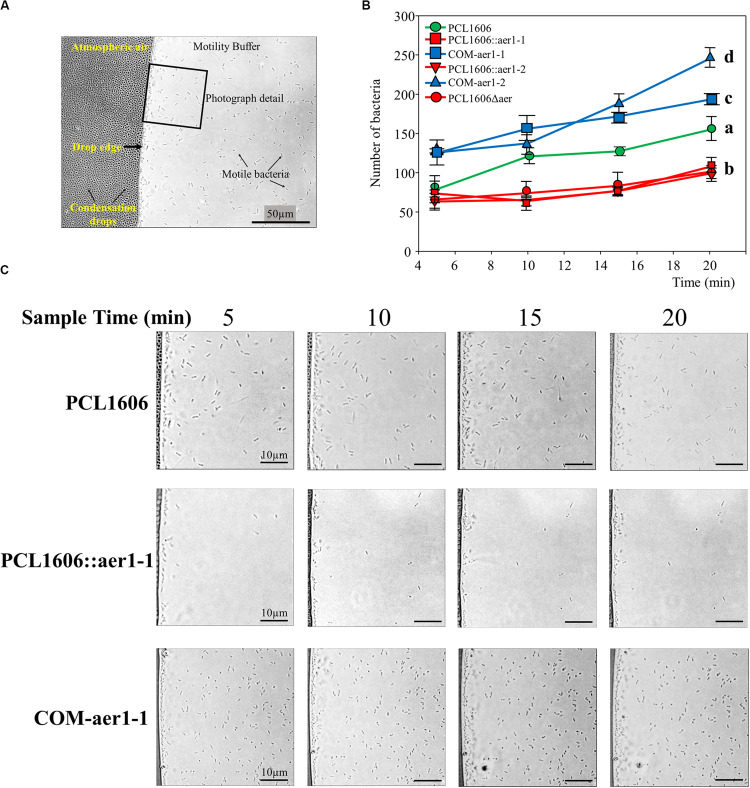
Optical microscope observations of bacterial behavior around the buffer drop in the presence of atmospheric air. **(A)** Photography obtained at 1000 × of magnification showing where the assay design was schematized. The images were taken at the drop edge where the bacteria were gathered. The atmospheric air side and buffer side are indicated, as is the drop edge, bacteria cells and small condensation drops in the coverslip. A 50 μm × 50 μm square section of each photograph is shown in section C. **(B)** Graph representation of the number of bacteria of each strain counted on microscopy photographs and located in the drop edge at 5, 10, 15, and 20 min. Wildtype *Pseudomonas chlororaphis* PCL1606 (PcPCL1606) strain is in green, defective insertion mutants and double-deletion mutants are in red, and complemented strains are in blue. The bacteria were placed approximately 7 μm from the drop edge, and the remainder of the bacteria was discarded. Letters indicate significant differences at the endpoint (20 min). Data analysis based on three photographs from three different fields was performed by one-way analysis of variance with Shapiro-Wilk normality test followed by all pairwise multiple comparisons (Tukey test) *p* < 0.05 (SigmaPlot 12.0). **(C)** Representative photographs of PcPCL1606, single-insertion mutant PCL1606::aer1-1 and its defective complemented strain COM-aer1-1 showing behavior after 5, 10, 15 and 20 min of air exposure. Sections of 50 μm × 50 μm from the original photograph are shown.

The air effect on the wildtype PcPCL1606 and single- and double-defective mutants was observed to be more precise at the microscopic level, where similar results were obtained (Figure 4). In this assay, the bacteria were at the edge of the drop in contact only with the air. PcPLC1606 had increased cell numbers over time. However, a reduced number of bacteria were observed when *aer*1-1 or *aer*1-2 was inactivated or both genes were absent. The reversed mutation by complementation with pLac2 or pLac4 replicant vectors led to a higher number of bacteria located in the drop edge, even more than generated by the wildtype strain (Figure 4). These results were obtained by insertion mutants and later confirmed by deletion mutants.

A similar microscopic experiment using control strains was performed to remove vector influences. Thus, PcPCL1606 and defective single-insertion mutants were transformed with pBBR1MCS-5 (Table 1), the cloning vector used in pLac2 and pLac4 construction. In addition, complementation vectors (pLac2 and pLac4) were inserted into the wildtype PcPCL1606 to phenotypically study the increase in Aer1-1 and Aer1-2 receptors. Wildtype PcPCL1606 and defective insertion mutants with an empty cloning vector did not show unexpected behavior. The wildtype cells with or without the empty cloning vector pBBR1MCS5 did not show significant differences. Defective insertion mutants, also transformed with pBBR1MCS5, showed significantly different expression in the wildtype PcPCL1606, as if they had not been transformed with a cloning vector. When pLac4 was inserted into wildtype PcPCL1606, a significant increase in bacteria at the edge drop was observed. This increase was also observed in the single mutant PCL1606::aer1-1 when complemented with pLac4. However, there were no significant differences between the wild type and wild type with pLac2, which had been cloned into the *aer*1-2 gene (Supplementary Figure S2).

### Influence of the Oxidizable Substrates in the Aer Transducers of PcPCL1606

Swimming motility in soft agar of all strains used in this study was analyzed in minimal medium M9 supplemented with one carbon source at 2 mM for each experiment (Supplementary Tables S3, S4). All defective strains, such as insertion mutants PCL1606::aer1-1 and PCL1606::aer1-2, deletion mutants PCL1606Δaer1-1, PCL1606Δaer1-2 and PCL1606Δaer, complemented strains COM-aer1-1 and COM-aer1-2, and control strains PCL1606pBBR, PCL1606pLac2, PCL1606pLac4, PCL1606::aer1-1pBBR and PCL1606::aer1-2pBBR, were compared with wildtype PcPCL1606, with the wildtype results given a value of one, and the defective strain data as normalized to the wildtype data.

**FIGURE 5:**
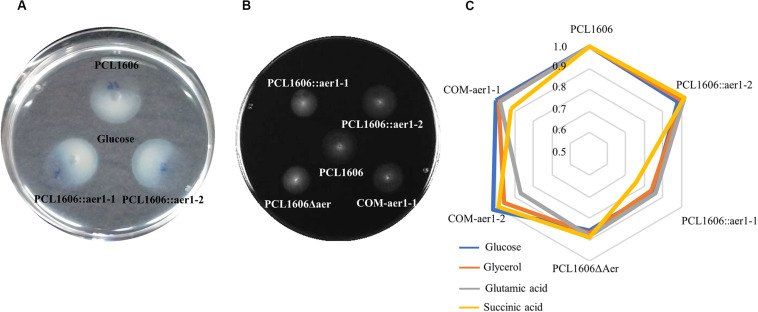
Swimming motility assays in soft agar were performed using a specific carbon source. **(A)** Chemotaxis of wildtype *Pseudomonas chlororaphis* PCL1606 (PcPCL1606) and single-insertion mutants PCL1606::aer1-1 and PCL1606::aer1-2 showing chemotaxis using glucose 2 mM as the attractant. **(B)** Swimming motility in soft M9 agar medium of defective single-insertion mutants PCL1606::aer1-1 and PCL1606::aer1-2, double-deletion mutant PCL1606Δaer, and complemented strain COM-aer1-1 compared with PcPCL1606. Diameter measurements were used for statistical analysis. Data were analyzed by Kruskal-Wallis test (one-way analysis of variance on ranks). Data were obtained from three independent experiments with three replicates for each. **(C)** Radar chart of normalized motility shown by wildtype PcPCL1606, the value of which was one, defective insertion mutants PCL1606::aer1-1 and PCL1606::aer1-2, double-deletion mutant PCL1606Δaer, and complemented strains COM-aer1-1 and COM-aer1-2, in the presence of the four carbon sources that presented significant differences: glucose in blue, glycerol in orange, glutamic acid in gray and succinic acid in yellow.

To confirm that the obtained results were not influenced by possible chemotaxis defects, chemotaxis assays using the same carbon sources as attractants were performed. In contrast to previous results, there were no differences between the wildtype and mutant strains in all cases (Figure 5A). According to the swimming motility results, only four substrates were shown to influence the gene *aer*1-1 mutants, which presented with less swimming motility than did wildtype PcPCL1606. These substrates were glucose, glycerol, glutamic acid, and succinic acid (Figures 5B,C), and these results were the same for both insertion, deletion, and double-deletion mutants (Supplementary Table S3). Defective *aer*1-2 genes, because of insertion or deletion mutations, did not show differences with respect to the wild type. When the mutated *aer*1-1 gene was complemented with pLac4, the motility of the mutant strain in the presence of these four carbon sources was equivalent to that of wildtype PcPCL1606.

Curiously, when there were no differences between the results of the wildtype and mutant strains;, that is, when the relative values of the colony diameter obtained from the mutants were close to one, the complemented mutants showed less motility than the wild type (Supplementary Table S3). This occurred for COM-aer1-1 and COM-aer1-2 in the presence of arabinose, fructose, galactose, sucrose, xylose, and malic acid, and for COM-aer1-2, it also occurred with glutamic acid. To clarify whether this effect is caused by the complementation method, swimming tests were performed with wildtype PcPCL1606 transformed with vectors pLac2 and pLac4 (Table 1). In this case, smaller colony diameters were obtained for all the carbon sources used for each vector (Supplementary Table S4).

### Study of Alternative Electron Acceptors to Oxygen

The signal perceived by Aer transducers originates through the energy production of the cell, such as changes in the electron transport system (ETS), the redox status of the cell and/or proton motive force (PMF), which are not the stimulus *per se*; then, energy taxis can be inferred. Thus, compounds that can substitute the oxygen in the ETS may be susceptible to any influence on the energy status when the oxygen is in low concentration and therefore are involved in energy taxis. Among these electron acceptors could be nitrate or dimethyl sulfoxide (DMSO).

The nitrate reduction test for PcPCL1606 and single-insertion mutants showed the same result independent of the anaerobiosis and aerobiosis environment. The Durham bell did not show gas from gas nitrogen production, and when Nit1 and Nit2 reagent was added, no red reaction was evident in the bacterial cultures, however, when zinc powder was added, the medium turned red, revealing the presence of nitrate in the bacterial cultures. Therefore, the results for the nitrate reduction test were negative for the wildtype PcPCL1606 and insertion mutants PCL1606::aer1-1 and PCL1606::aer1-2.

On the other hand, chemotaxis assays using nitrate and DMSO as attractive substances in anaerobic and aerobiotic environments were performed, even though nitrate reduction in the standard test had been negative. No movement was observed toward nitrate solution or DMSO from any strain used, including wildtype PcPCL1606 and the defective insertion mutants. The controls in the aerobiotic incubation showed radial development around the inoculation point of every strain. Therefore, the results from the assay of chemotaxis to nitrate and DMSO, used as alternatives to oxygen, were also negative.

### Role of Aer Transducer Receptors in the Biology of PcPCL1606

**FIGURE 6:**
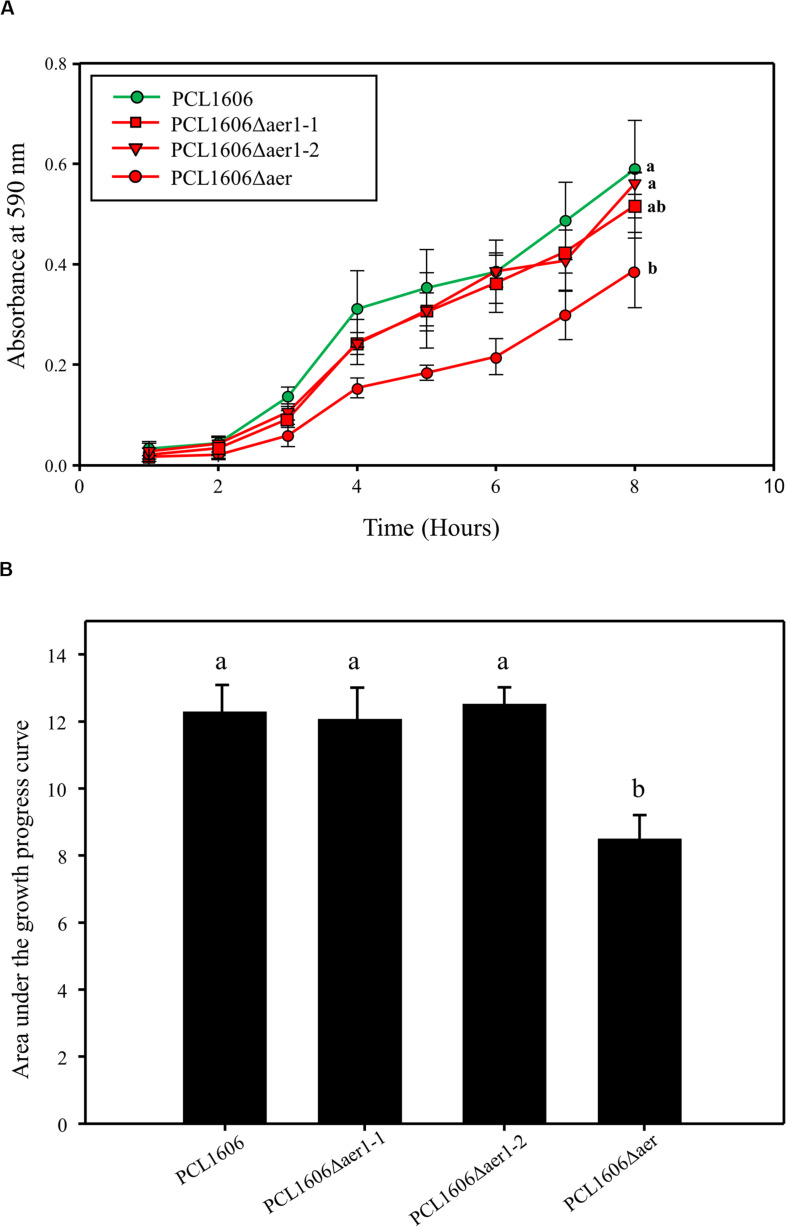
Bacterial adhesion to the glass surface was quantified by the crystal violet method. **(A)** Adhesion 8-hour monitoring of wildtype *Pseudomonas chlororaphis* PCL1606 (PcPCL1606), single-deletion mutants PCL1606Δaer1-1 and PCL1606Δaer1-2, and double-deletion mutant PCL1606Δaer. Letters indicate significant differences at the endpoint. **(B)** Area under the growth progress curve showing adhesion after 24 h of monitoring PcPCL1606 and the single- and double-deletion mutants. Letters indicate significant differences obtained by one-way analysis of variance with a Shapiro-Wilk normality test, followed by all pairwise multiple comparison procedures (Tukey test) *p* < 0.05 (SigmaPlot 12.0).

Assays of swarming motility and adhesion to the glass surface were performed to determine Aer transducer involvement in the colonization process. Swarming motility experiments showed no significant differences between the wildtype and defective deletion mutants (data not shown). However, adhesion experiments showed a slight deficiency in the defective single-deletion mutants compared with that of wildtype PcPCL1606, but this difference was not significant; only the double-deletion mutant, PCL1606Δaer, presented with a significant disadvantage to glass adhesion in an 8 h timeframe (Figure 6A). This lower adhesion of the double mutant was also observed in a 24 h period, while single-deletion mutants seemed to show wildtype-like behavior after one day (Figure 6B).

**FIGURE 7:**
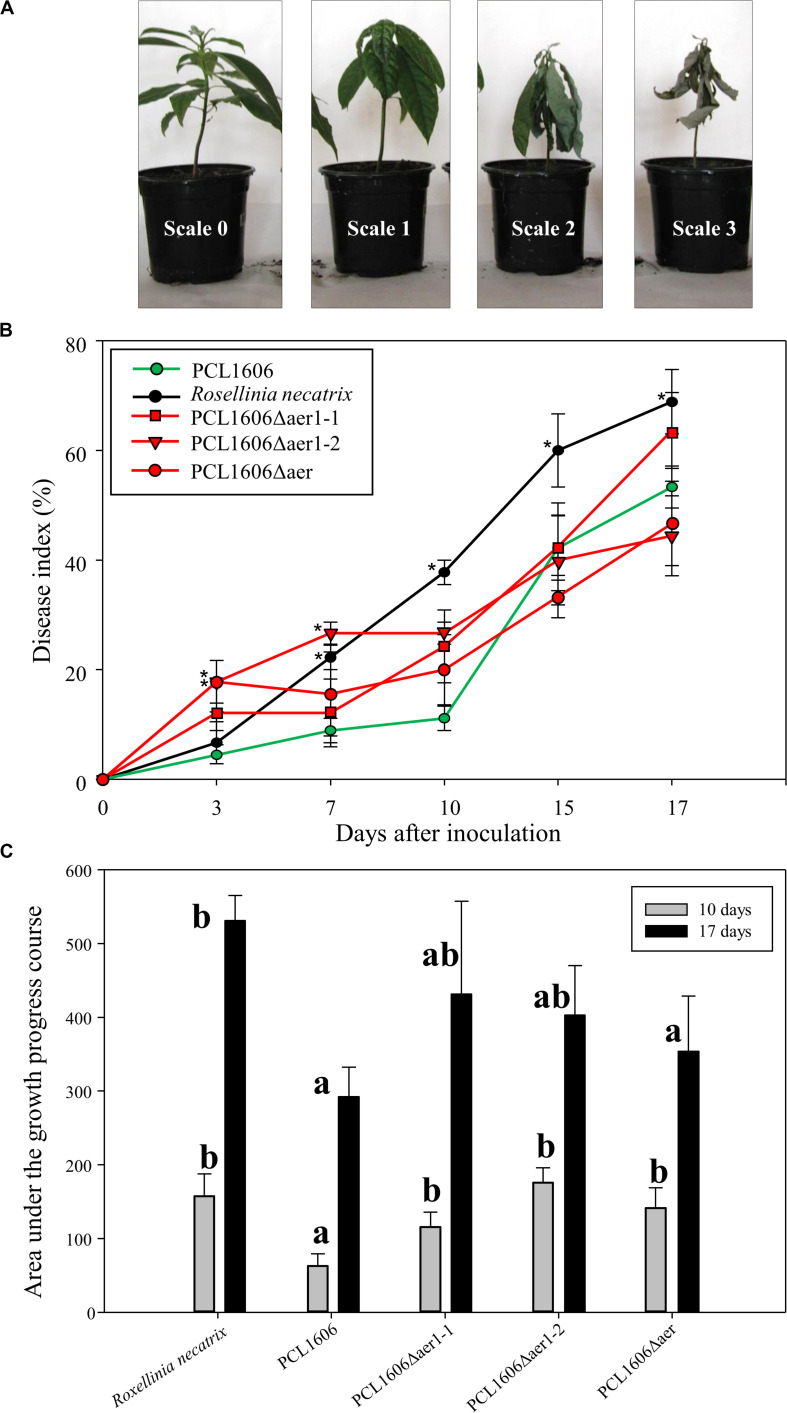
Biocontrol exhibited by *Pseudomonas chlororaphis* PCL1606 (PcPCL1606), single-deletion mutants PCL1606Δaer1-1 and PCL1606Δaer1-2 and double-deletion mutant PCL1606Δaer against *Rosellinia necatrix* during avocado root infection. **(A)** Representative photographs of disease on avocado white root rot, scaled from 0 to 3, on 6-month plants. The number of plants in each state of the scale was used in the calculation of the disease index. **(B)** Time course representation of the disease index of the avocado plants infected by *R. necatrix* and inoculated with wildtype PcPCL1606 (green line) and single- and double-deletion mutants (red lines). Infected plants without bacterial inoculation were used as negative controls (black line). The graph shows five symptom test points 3, 7, 10, 15, and 17 days after inoculation. Points with asterisks indicate the disease index with significant differences compared to wildtype PcPCL1606. **(C)** Area under growth progress curve shown in section B for the PcPCL1606, single- and double-deletion mutants and *R. necatrix* after 10 days (gray bars) and 17 days (black bars). Letters indicate significant differences. Statistical analysis by *T*-test with α = 0.05 and 4 degrees of freedom (SigmaPlot 12.0).

The participation of Aer transducers in the biocontrol process of PcPCL1606 was analyzed by bacterial inoculation of avocado roots and infection with *Rosellinia necatrix*. The results shown in the disease index indicated a clear difference between the presence and absence of wildtype PcPCL1606 (Figure 7). The lack of *aer*1-1, *aer* 1-2 or both resulted in a delay in biocontrol activity, and symptoms such as *R. necatrix* infection were observed in the first 10 days of the experiment. However, during the remaining 7 days, the biocontrol activity led to symptom levels close to those of wildtype PcPCL1606 (Figure 7B). This behavior of the Aer mutants suggests the possible participation of Aer receptors in the early stages of root colonization by an antagonist (Figure 7C).

**FIGURE 8:**
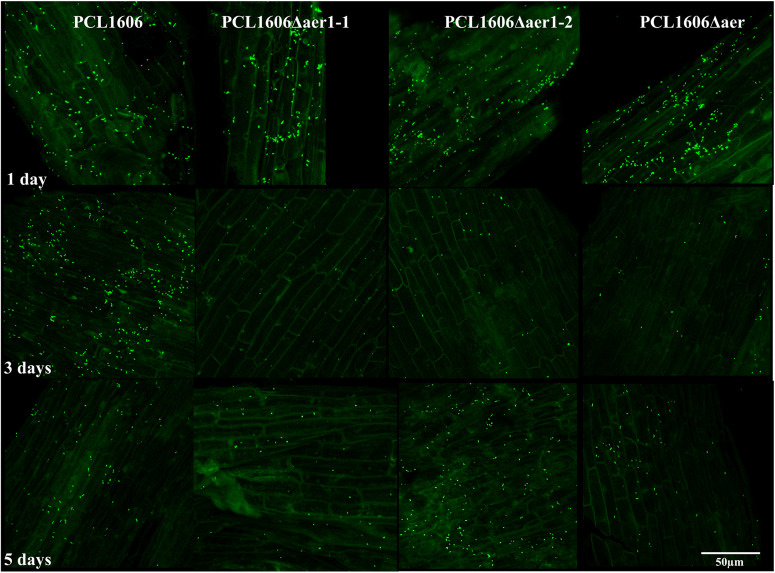
*In situ* visualization of the GFP-tagged bacteria colonized at the root tip in a short-term experiment, as observed by confocal laser-scanning microscopy. One-month-old roots from avocado plants were disinfected and maintained in a gnotobiotic system under controlled conditions. One milliliter of bacterial suspension, adjusted to an OD_600_ of 1, was inoculated in the neck of the plant, and bacterial colonization progress was observed. Photographs were taken at 630 × magnification at 1, 3, and 5 days after inoculation of *Pseudomonas chlororaphis* PCL1606GFP, single-deletion mutants PCL1606Δaer1-1GFP and PCL1606Δaer1-2GFP, and double-deletion mutant PCL1606ΔaerGFP, marked with green fluorescent protein.

**FIGURE 9:**
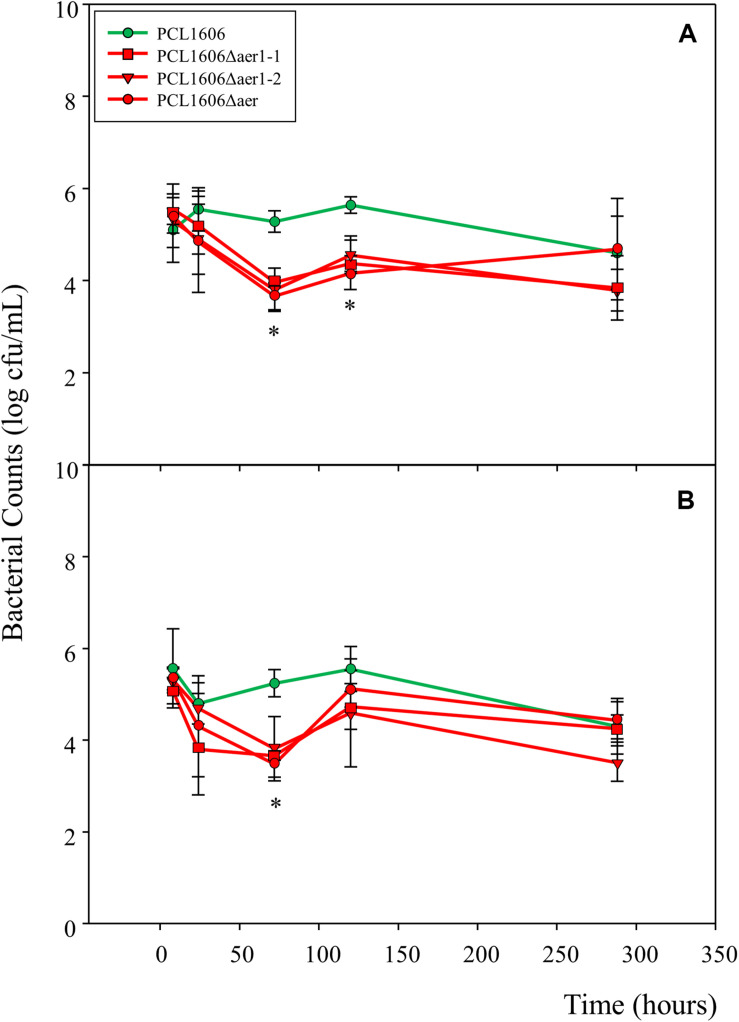
Number of bacterial colonies and measure of persistence on the avocado rhizosphere. Root samples from plants maintained in sterile vermiculite and controlled conditions were taken at 8 h, 24 h (1 day), 72 h (3 days), 120 h (5 days) and 288 h (12 days). *Pseudomonas chlororaphis* PCL1606 (PcPCL1606) (green), single-deletion mutants PCL1606Δaer1-1 and PCL1606Δaer1-2 and double-deletion mutant PCL1606Δaer (red) strains have been used. **(A)** Colonization was analyzed at the root tips (sections of 1–1.5 cm at the tip). **(B)** Persistence was studied on root sections 2–6 cm from the root tips. Data were analyzed for significance using one-way analysis of variance with a Shapiro-Wilk normality test, followed by all pairwise multiple comparisons (Tukey test), *p* < 0.05 (SigmaPlot 12.0). The asterisk marks a significant difference between the single- and double-deletion mutants and wildtype PcPCL1606.

A short-term experiment of colonization in avocado roots in a gnotobiotic system was performed (Figure 8). Observations of green-marked strains after 24 h of colonization revealed the equivalent abundance of wildtype and mutant bacterial strains, and no relevant differences were evident. However, after 3 days, the defective mutants nearly disappeared, while the wildtype PcPLC1606 strain maintained the same level on the avocado root. Two days later, the bacterial levels seemed to slowly recover (Figure 8). Due to the destructive nature of processing the samples in this test, it is necessary to corroborate the results with complementary experiments. Thus, bacteria were counted over 12 days (Figure 9) both at the tip of the roots and in distal areas. On the basis of the colonization, the bacterial settlement in the tip of the root (Figure 9A), and bacterial survival or persistence, the bacteria were found in the distal area (Figure 9B). In terms of both colonization and persistence, the level of deletion-mutant bacteria decreased by two orders of magnitude compared with wildtype PcPCL1606 on the third day. Mutant bacteria levels recovered on the fifth day, reaching levels equivalent to those of the wildtype strain by day twelve.

## Discussion

### PcPCL1606 Has Two *aer* Genes

Energy taxis, or aerotaxis, as it is more commonly known, has been widely studied in enterobacteria such as *Salmonella enterica* serovar Typhimurium (Taylor, 1983) or *Escherichia coli* (Rebbapragada et al., 1997; Bibikov et al., 2000); the latter has been the preferred model because of the simplicity of its signal receptor system. From these studies, Aer receptors have been detected successively in more bacterial species from the most diverse environments, such as *Pseudomonas putida* (Nichols and Harwood, 2000), *Pseudomonas aeruginosa* (Hong et al., 2004), *Shewanella oneidensis* (Baraquet et al., 2009) and *Azospirillum brasilense* (Xie et al., 2010). The model rhizobacterium PcPCL1606 showed two loci with corresponding Aer features, PCL1606_41090 (gene *aer*1-1) and PCL1606_20530 (gene *aer*1-2) among the thirty-one putative MCPs detected. Aer transducers are representative of the PAS domain-containing receptor MCP family, the members of which do not have a periplasmic domain but face the cytoplasm and are linked to the membrane by an anchor of hydrophobic amino acids (Amin et al., 2006; Taylor, 2007). The PAS domain superfamily is very diverse in various life forms and includes transducer molecules that can sense light, oxygen or redox potential as input signals (Taylor, 2007). The *aer*1-1 and *aer*1-2 genes are in different genome locations, although they have the same putative function and high sequence similarity; therefore, they are two independent genes.

The mode of action of Aer receptors or transducers implies that they are linked to FAD, which is a cofactor that guides the bacteria in a gradient of rapidly oxidizable substrates (Greer-Phillips et al., 2003). The Aer-dependent tactic response was experimentally linked to redox changes in the cell since the PAS domain and FAD binding are essential for Aer function (Edwards et al., 2006). In the *E. coli* Aer model, PAS-FAD reacted to changes in the cellular redox state by interacting either with a cytosolic electron donor or directly with the electron transport chain. Further, a role for NADH dehydrogenase I in Aer-based energy sensing, possibly through protein-protein interactions, was detected (Edwards et al., 2006). When an energy taxis signal is transduced via a direct interaction of the PAS domain, the resulting conformational change affects the signaling ability of the MCP domain in the protein, and then, Aer interacts with other MCPs in chemoreceptor arrays (Schweinitzer and Josenhans, 2010). PAS domain-containing receptors can be classified into three major groups based on their sequence conservation (Xie et al., 2010). Class I Aer transductors are embedded in the membrane, and class II transductors are free in the cytoplasm; both types bind FAD non-covalently and are exemplified by *E. coli* Aer and *A. brasilense* AerC, respectively. The third class comprises unrelated PAS domain-containing receptors, which may not bind FAD. Based on domain structure and transmembrane region, as well as their sequence identity with Aer receptors, Aer1-1 and Aer1-2 seem to belong to class I. Most Aer receptors found in *Pseudomonas* species belong to this class, according to the bioinformatics analysis published by Booth and Turner (2016). Aer class I receptors are described as encoded by the ancestral gene and are the most prevalent in all the *Pseudomonas* species used in the study (Booth and Turner, 2016). These authors detected sequence duplicity in few of the species analyzed that did not share synteny with the other members of class I Aer transducers. In the current study, paralogous *aer* genes were analyzed in the rhizobacterium model *Pseudomonas chlororaphis*. We found that both genes were grouped separately, resulting in *aer*1-1-like and *aer*1-2-like genes (except protein AZD68398.1 of *P. chlororaphis* subsp. *aurantiaca* strain DSM19603), suggesting early *aer* gene duplicity in this species.

Almost all ancestral *aer* class I genes, including *aer*1-1, share synteny and are associated with an aconitate gene. Aer cannot obtain electrons for reducing the FAD cofactors through aconitase because the isomerization of citrate to isocitrate is a non-redox process, suggesting that isocitrate dehydrogenase may be the step in the citric acid cycle that is sensed during energy taxis. On the other hand, CAAX amino protease may not be involved in their functioning (Booth and Turner, 2016). The other two genes near *aer*1-1 were alpha/beta hydrolase and cytochrome C oxidase. The first is in a superfamily of hydrolytic enzymes of broad phylogenetic origin and catalytic function on the same order of magnitude (Ollis et al., 1992), and cytochrome C oxidase is a large transmembrane protein complex that forms the final step in the respiratory electron transport chain located near the membrane of cells, converting the molecular oxygen into two molecules of water (Castresana et al., 1994), suggesting that both enzymes could be involved in the redox process. The location of the duplicated gene *aer*1-2 is slightly more varied and seems to be related to aerotaxis, in the sense that the enzyme it encodes is involved in the transformation of substrates to obtain energy.

### Aer Receptors Are Functional in PcPCL1606

Aerotaxis is a response to changes in respiratory electron flow that result from an increase or decrease in oxygen concentration; in the absence of oxygen, alternative electron acceptors such as fumarate or nitrate support electron flow (Taylor et al., 1999). According to air trap and drop assay experiments, PcPCL1606 showed a strong attraction to atmospheric air, explaining the mobility that moves bacteria from outside to inside the Pasteur pipette, presumably attracted by the availability of atmospheric air. This attraction was dramatically decreased when *aer*1-1, *aer*1-2 or both were inactive. This finding revealed the requirement of both genes for full taxis to air. However, PcPCL1606 does not seem to use nitrate or DMSO as an alternative to oxygen, although putative nitrate and nitrite reductase functions are present in its genome. The predilection of PcPCL1606 for atmospheric oxygen was also demonstrated by the bacterial-band location (Tamar et al., 2015) in the medium column inside the Pasteur pipette, which was always located at the top and in contact with air, in the first line of gas exchange when both *aer* genes were functional. An additional argument to understand the dependence of PcPCL1606 of the oxygen, is the inability to produce phenazines and therefore the absence of it. Glasser et al. (2014) reported that phenazine enable the proton-motive force by stimulate redox homeostasis and ATP synthesis, maintaining the bacterium survival in anaerobic environmental (Glasser et al., 2014; Bosire and Rosenbaum, 2017; Askitosari et al., 2019). The non-production of phenazine by PcPCL1606 prevents it from having this resource to survive in oxygen-limiting conditions, and makes it a perfect candidate for the study of *aer* genes without the interference of these redox compounds (Hernandez et al., 2004; Wang and Newman, 2008).

The taxis to the air was confirmed by direct optical microscope observations in the drop-assay experiment. Within minutes, the cell headed to the interface group around the drop edge when Aer1-1 and Aer1-2 were present. However, when one or both was absent, the movement was more erratic, and the bacteria movement to the air-liquid interface was much lower. However, none actively moved away from the drop edge, as was described by Bibikov et al. (1997) for *Escherichia coli aer* mutants. In complemented strains, the bacterial counts increased, as observed in the drop edge, according to the Aer-transducer increment. These findings indicate a direct relationship between Aer1-1 and Aer1-2 and their attraction to atmospheric air.

Other compounds affecting energy taxis can be metabolizable substrates, e.g., carbon or nitrogen sources, such sugars, amino acids and organic acids. The ability to sense altered metabolism, as is the case in energy taxis, provides a very efficient means of monitoring the bacterial environment and integrating different stimuli that impact energy levels, which are initially the same as the level of the sensor (Schweinitzer and Josenhans, 2010). Of all metabolites used, only glucose, glycerol, glutamic acid, and succinic acid showed differences when Aer1-1 was absent. Aer1-2 seem to be unable to sense energy fluctuations linked to metabolism. These four substrates are composed of monosaccharides (glucose), the principal sugar for obtaining energy in ATP form and pyruvate by the Krebs cycle. Pyruvate can be oxidized, producing NADH and FADH_2_. An alcohol (glycerol) that can be modified to pyruvate, such as glucose, is involved in the Krebs cycle. An amino acid (glutamic acid), a component of root exudate from plants (de Weert et al., 2002) that can be oxidatively deaminated by glutamate dehydrogenase, is the only known enzyme that can interact with both NAD and NADP as a redox coenzyme. Oxidation is thought to occur with the transfer of a carbon hydride ion from glutamate to NAD(P), forming α-iminoglutarate, which is hydrolysed to α-ketoglutarate and ammonium. The α-ketoglutarate product has been regenerated by oxidative deamination to become a product of transamination. According to the needs of the cell, it can also be used in the Krebs cycle. Finally, an alkane (succinate), another component of root exudate from plants (de Weert et al., 2002) and which oxidation to fumarate can reduce FAD to FADH_2_ (Nelson and Cox, 2017). The response to oxidable substrates is dependent on the presence of the FAD cofactor in association with Aer, which is consistent with the sensing mechanism (Greer-Phillips et al., 2003). Therefore, these four substrates, glucose, glycerol, glutamic acid, and succinic acid, are involved in redox pathways that directly affect the Aer signaling domain of Aer1-1, and Aer1-2 seems to function independent of this signaling pathway.

Further, significant differences were observed in complemented strains when redox changes were induced through metabolizable substrates. This result can be explained by an uncontrolled increase in Aer receptors that seems to affect the tactic mechanism of the MCPs. As the bacteria consume the substrate, a gradient is created such that both Aer and chemotaxis receptors engage in the mobility process. In the presence of MCPs, Aer, a receptor with low abundance, has little control over the steady-state swimming pattern of a cell. Methylation enables the MCPs to adjust the swimming pattern to elicit optimal chemotactic responsiveness (Bibikov et al., 2004). The increase in Aer transductors can cause saturation that affects the methylation-demethylation rate and thus swimming motility. However, Aer-mediated aerotaxis is based on methylation independent of CheR or CheB (Bibikov et al., 2004); therefore, o more Aer receptors should not affect the methylation of MCP receptors. On the other hand, the Aer signaling domain resembles that of conventional methyl-accepting receptors and, similar to that of MCPs, appears to be capable of regulating CheA autophosphorylation, presumably in a ternary complex with the CheW coupling protein (Bibikov et al., 2004). It is necessary to remember that only one functional *che*A gene has been identified in PcPCL1606 (Polonio et al., 2017). Therefore, an excessive increase in Aer transducers could affect the phosphorylation-dephosphorylation of the movement translation machinery, thus affecting the movement pattern.

### Aer Transductor Role in Interactions With Plants

In *Pseudomonas* species, chemotaxis was shown to be important for promoting plant growth and plant or animal infection (Kato et al., 2008; Tamar et al., 2015). We speculate that energy taxis is important for the dynamic responses of *P. chlororaphis* to its diverse natural habitats. In this sense, single and double *aer* mutant and wildtype strains were tested in a biocontrol system, where plant roots, microorganisms and pathogens interact. A late response to disease control was observed by the *aer* mutants, recovering standard behavior after the first 10 days, quite possibly by 2-hexyl 5-propyl resorcinol (HPR) production, a powerful antibiotic produced by PcPCL1606 that inhibits pathogen development (Cazorla et al., 2006; Calderón et al., 2014). This late response could be related to a disturbance of first settlement and colonization of the root by the *aer* mutants. The *aer* mutants tested *in vitro* did not show differences in swarming movement, and only the double *aer* mutant showed an effect in adhesion to the glass surface. However, single *aer* mutants showed a delay in the biocontrol assay; therefore, *in vitro* experiments may not have shown *aer*1-1 and *aer*1-2 mutant deficiencies. When *in vivo* adhesion and colonization properties were tested in avocado roots using an axenic system, non-stable settlement was observed for the *aer* mutants, in contrast to that exhibited by wildtype PcPCL1606, the presence of which was more or less consistent over 5 days. This behavior was confirmed through bacterial counts determined during the root colonization and persistence tests. In these assays, wildtype PcPCL1606 maintained similar levels throughout the experiment, while there was a decrease in the number of mutants from the third day of the experiment, with progressive recovery in the following days.

Linking the findings obtained *in silico*, *in vitro*, and *in vivo* in the current research and integrating them with previously acquired knowledge of PcPCL1606, it is easy to conclude that movement capacity, the chemotaxis system and energy requirements are essential for making avocado roots the preferred niche. Avocado plants have abundant branched roots located in the first meter of soil (Cordero and Boshier, 2003), where the greatest amount of oxygen accumulates, since it is the area where gases are freely exchanged. In previous publication, the attraction of PcPCL1606 for avocado root and *Rosellinia necatrix* exudates was confirmed (Polonio et al., 2017), and the dependence of this interaction on oxygen was tested in the present work. Accordingly, this antagonist is initially attracted to healthy and fungus-infected avocado roots. Once the attractants are detected, the bacteria moves to the rhizoplane.

In the point of movement to the rhizoplane, the Aer receptor plays an important role, confirming this niche as profitable by redox potential measures, settling the bacteria on the root and contributing to the change from mobile life to sessile life, thus increasing the bacterial presence at the plant roots and supporting the quorum sensing process as well as antibiotic production. When Aer receptors are absent, then the bacteria is attracted by root exudate, but this niche is not recognized as a preferred zone, and then, the bacteria, still in mobile life, are lost, looking around for oxidizable metabolites and oxygen. That does not mean that the bacteria will be mobile forever, PcPLC1606 shows high amounts of MCPs and PAS domain proteins, which may detect shortcomings in energy inside the cytoplasm, but likely with less efficiency, thus resulting in a delay in the settlement process and assuming more risks to their survival.

## Data Availability Statement

The original contributions presented in the study are included in the article/Supplementary Material, further inquiries can be directed to the corresponding author.

## Author Contributions

FC led the project, procured funding and took part in the experiments design, and manuscript structure. EA was involved in the experiments design, conducting the experiments, collecting data, designing figures, tables, and writing the manuscript. All authors reviewed the manuscript and approved the final version.

## Conflict of Interest

The authors declare that the research was conducted in the absence of any commercial or financial relationships that could be construed as a potential conflict of interest.

## Footnotes

1 https://www.ebi.ac.uk/Tools/hmmer/search/phmmer

2 http://www.softberry.com

3 http://www.enzim.hu/DAS/DAS.html

4 https://www.ebi.ac.uk/Tools/msa/clustalo/

5 www.endmemo.com
