# Supplementary material for: Aer Receptors Influence the Pseudomonas chlororaphis PCL1606 Lifestyle
Source: Front Microbiol. 2020 Jul 8;11:1560. doi: 10.3389/fmicb.2020.01560 (PMC7367214; doi:10.3389/fmicb.2020.01560)
Supplement: Supplementary file 1 [file Data_Sheet_1.PDF]

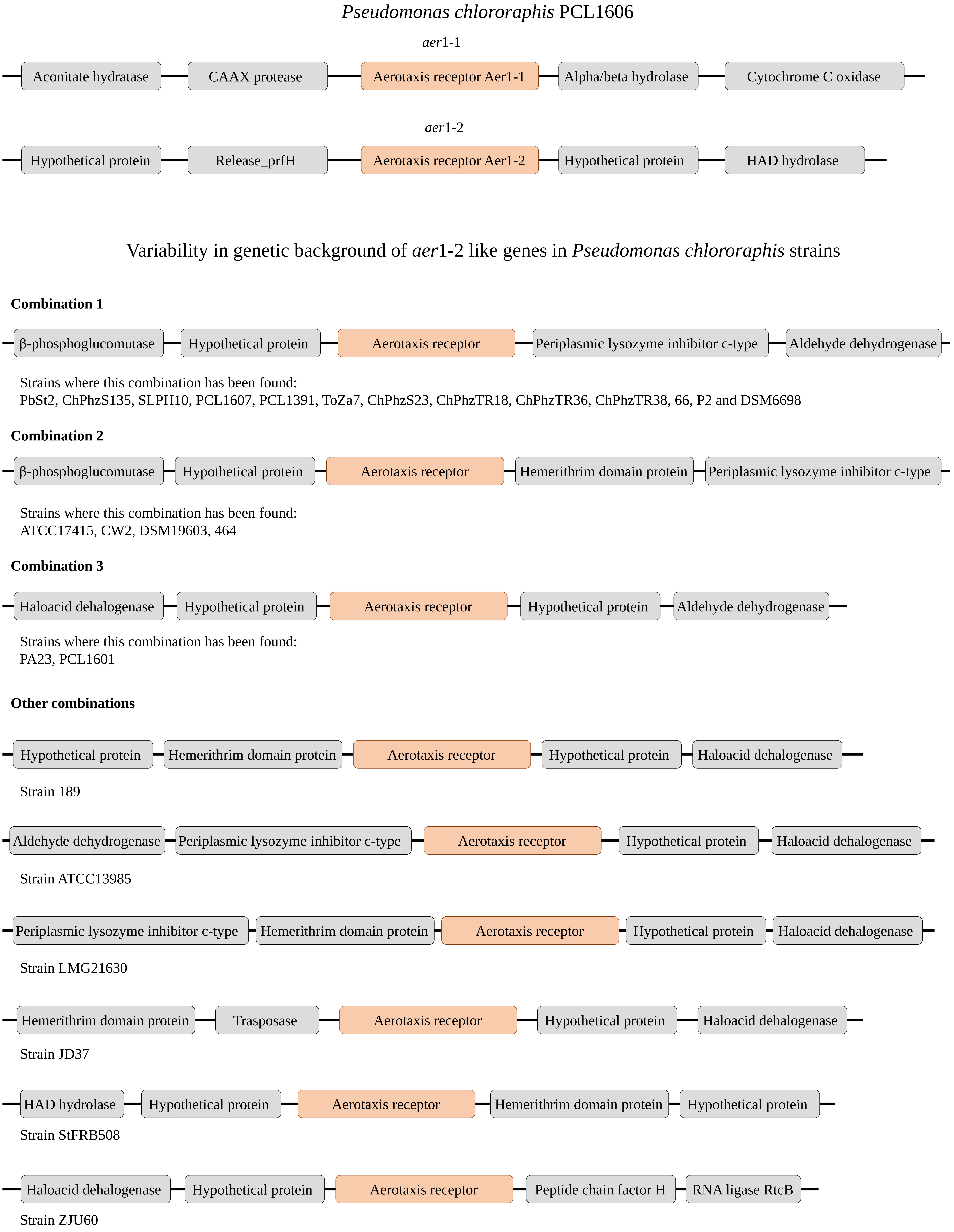

Supplementary Figure S1. The genetic background of the putative aerotaxis receptor genes (*aer*1-1 and *aer*1-2) found in PcPCL1606, with putative functions of two coding sequences (CDS) upstream and two CDS downstream as indicated. Different combinations in the genetic background of the *aer*1-2-like genes found in *Pseudomonas chlororaphis* strains (**Supplementary Table S1**) are also indicated.
