# Supplementary material for: Aer Receptors Influence the Pseudomonas chlororaphis PCL1606 Lifestyle
Source: Front Microbiol. 2020 Jul 8;11:1560. doi: 10.3389/fmicb.2020.01560 (PMC7367214; doi:10.3389/fmicb.2020.01560)
Supplement: Supplementary file 2 [file Data_Sheet_2.PDF]

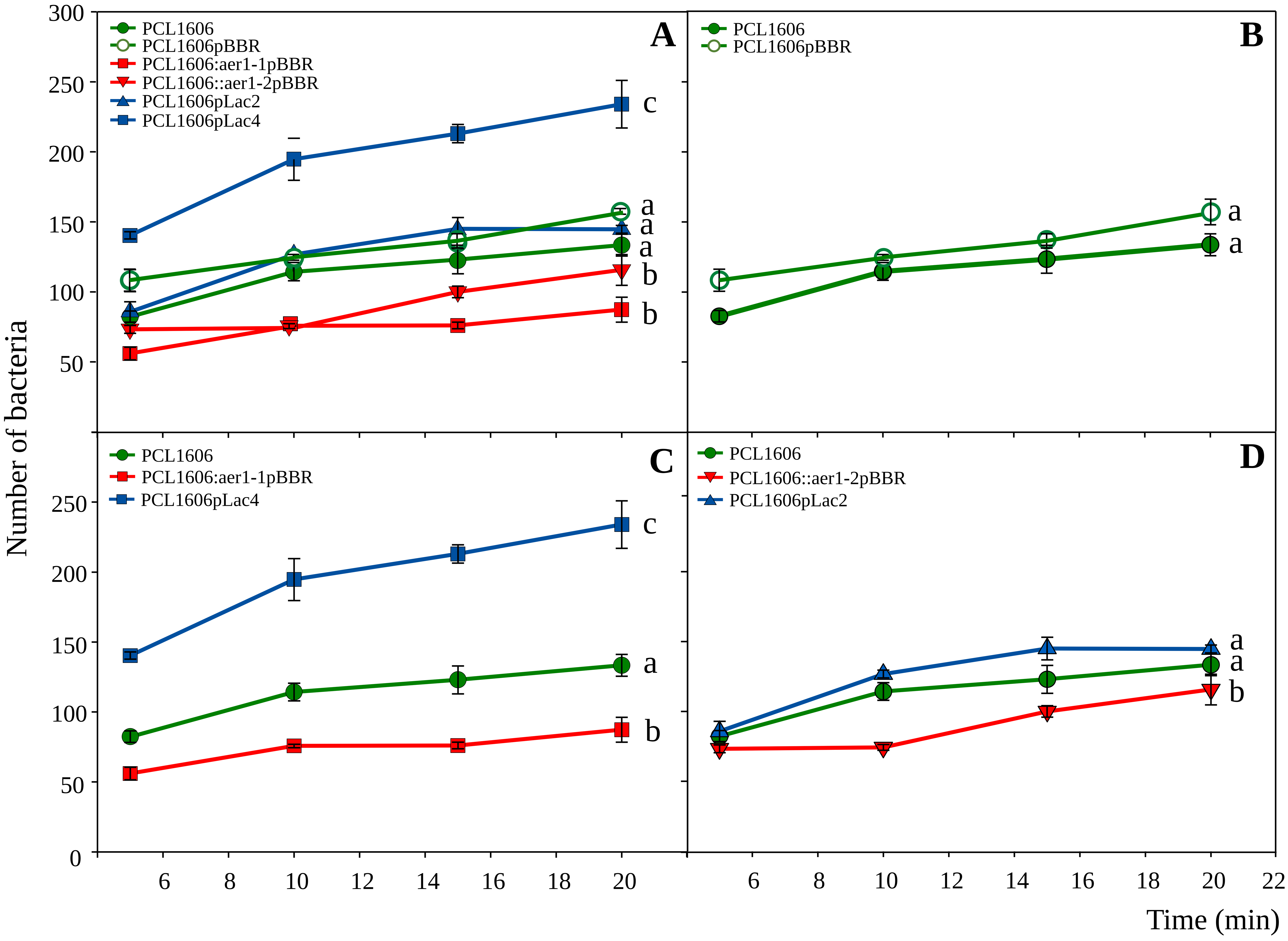

Supplementary Figure S2. Bacterial counts in the drop edge of the buffer in direct contact with atmospheric air at 5, 10, 15 and 20 min were analysed by optical microscope observations. A) Joint graph representation of the number of cells located at the drop edge of all control strains at the sampling times indicated. *Pseudomonas chlororaphis* PCL1606 and PCL1606 transformed with pLac2 (PCL1606pLac2), pLac4 (PCL1606pLac4) and pBBR1MCS-5 (PCL1606PBBR). Single-insertion mutants PCL1606::aer1-1 and PCL1606::aer1-2 were also transformed with the empty vector pBBR1MCS-5 (PCL1606::aer1-1PBBR and PCL1606::aer1-2PBBR). B) Graph representation of only PCL1606 with empty vector pBBR1MCS-5, compared with wildtype *P. chlororaphis* PCL1606. C) Graph representation of only PCL1606 with pLac4 and insertional mutant PCL1606::aer1-1 with empty vector pBBR1MCS-5, both compared with wildtype *P. chlororaphis* PCL1606. D) Graph representation of only PCL1606 with pLac2 and insertion mutant PCL1606::aer1-2 with empty vector pBBR1MCS-5, both compared with wildtype *P. chlororaphis* PCL1606. Letters indicate significant differences at the endpoint (20 min). Statistical analysis by T-test with $\alpha=0.05$ and 4 degrees of freedom (SigmaPlot 12.0).
