# Supplementary material for: Aer Receptors Influence the Pseudomonas chlororaphis PCL1606 Lifestyle
Source: Front Microbiol. 2020 Jul 8;11:1560. doi: 10.3389/fmicb.2020.01560 (PMC7367214; doi:10.3389/fmicb.2020.01560)
Supplement: Supplementary file 3 [file Table_1.DOCX]

Table S2. DNA primers used in this study.

| **PRIMERS** | **SEQUENCE 5’-3’** | **CHARACTERISTICS** |
| --- | --- | --- |
| **Construction of insertional mutants using integrative vector** | | |
| Mut20530F | CTGGCGACTTCCTGGGTG | Product size 730bp of the locus PCL1606_20530 |
| Mut20530R | ATATCGTCCGCCATCCCG |  |
| Mut41090F | CTACCAACGCATCAACCAGG | Product size 546bp of the locus PCL1606_41090 |
| Mut41090R | CGGTTGGCTTCCTGGGTG |  |
| **Construction of deletion mutants for cleanly gene remove** | | |
| Gb20530up-F | tctgaattcgagctcggtacccggg  GGGGGTGCTGCTGATGATT  CTGTTGCAGC | Product size 719bp of PCL1606_20520 located up stream of PCL1606_20530 gene |
| Gb20530up-R | gagcaatctggaaCGGCGAGG  CGCTGCGCACCTGACAT |  |
| Gb20530dw-F | cagcgcctcgccgTTCCAGATTG  CTCCTTGTGTCCTTTCAAG | Product size 655bp of PCL1606_20540 located down stream of PCL1606_20530 gene |
| Gb20530dw-R | gcatgcctgcaggtcgactctagag  GGCTCTTGAAGATAGCGC  CCCGCCT |  |
| Gb41090up-F | tctgaattcgagctcggtacccg  ggGCTGATTCCATTCTTTTCGC  AACAA | Product size 1002bp of PCL1606_41080 located up stream of PCL1606_41090 gene |
| Gb41090up-R | ttattgcctgggtGGTTTTTCTAT  CCGCAAGGCTTTGA |  |
| Gb41090dw-F | ggatagaaaaaccACCC  AGGCAATAAAAAACCCG  GAAGGCCC | Product size 932bp of PCL1606_41100 located down stream of PCL1606_41090 gene |
| Gb41090dw-R | gcatgcctgcaggtcgact  ctagagCGCACGGCAAG  GCGCGCTCACTTCT |  |
| **Complementation of *aer* genes by replicative plasmid** | | |
| COM20530F | AAAAGGATCCCAACCTTGA  ACCCCGCAAC | Product size 1831bp including complete ORF of PCL1606_20530 and two restriction points, BamHI in forward primer and HindIII in reverse primer, for correct orientation |
| COM20530R | AAAAAAGCTTCCGATGGCTT  GAAAGGACAC |  |
| COM41090F | AAAAGAATTCCTCGGCTTT  CTGGTTCTTCG | Product size 1836bp including complete ORF of PCL1606_41090 and two restriction points, EcoRI in forward primer and BamHI in reverse primer, for correct orientation |
| COM41090R | AAAAGGATCCGACGATGC  TGACTGACACAC |  |
