# Supplementary material for: Aer Receptors Influence the Pseudomonas chlororaphis PCL1606 Lifestyle
Source: Front Microbiol. 2020 Jul 8;11:1560. doi: 10.3389/fmicb.2020.01560 (PMC7367214; doi:10.3389/fmicb.2020.01560)
Supplement: Supplementary file 4 [file Table_2.DOCX]

Table S1. Codes and accession numbers of the genetic sequences used in consensus phylogeny tree, showed in Figure 2. The sequences are grouped according with highest similarity at nucleotides level of both loci found in *Pseudomonas chlororaphis* PCL1606 assembled chromosome (PCL1606_20530 and PCL1606_41090, respectively). Percentage of coverage and identity have been obtained by blastn comparison.

|  |  |  | **Nucleotic Sequences similar to PCL1606_20530** | | | | **Nucleotic Sequences similar to PCL1606_41090** | | | |
| --- | --- | --- | --- | --- | --- | --- | --- | --- | --- | --- |
| Bacteria | Strain | Accession number | Locus_tag | Protein id | Coverage % | Identity | Locus_tag | Protein id | Coverage % | Identity |
| **Genus *Pseudomonas*** | | |  |  |  |  |  |  |  |  |
| *Pseudomonas sp* | R1-43-08 | CP027734.1 |  |  |  |  | C4J87_3655 | AZF43797.1 | 99 | 86.18 |
| *Pseudomonas sp* | R3-52-08 | CP027730.1 |  |  |  |  | C4J91_3971 | AZF22704.1 | 99 | 86.62 |
| *Pseudomonas sp* | R4-34-07 | CP027760.1 |  |  |  |  | C4J85_3921 | AZF54389.1 | 99 | 86.30 |
| *Pseudomonas sp* | CMR12a | CP027706.1 |  |  |  |  | C4K39_2027 | AZC23711.1 | 99 | 88.24 |
| *Pseudomonas sp* | LBUM920 | CP027762.1 |  |  |  |  | C4J83_4421 | AZF65393.1 | 99 | 85.79 |
| **Species of *Pseudomonas*** | | |  |  |  |  |  |  |  |  |
| *Ps. orientalis* | R4-35-08 | CP027726.1 |  |  |  |  | C4J95_3844 | AZF01289.1 | 99 | 87.08 |
| *Ps. chlororaphis* | B25 | CP027753.1 |  |  |  |  | C4K04_2213 | AZE47896.1 | 99 | 94.25 |
| *Ps. chlororaphis*^1^ | PCL1606 | NZ_CP011110.1 | PCL1606_20530 | WP_044464188.1 | 100 | 100 | PCL1606_41090 | WP_044462163.1 | 100 | 100 |
| *Ps. chlororaphis* | Pb-St2 | CP027716.1 | C4K23_4048 | AZD30789.1 | 100 | 91.51 | C4K23_1910 | AZD28670.1 | 99 | 94.37 |
| *Ps. chlororaphis* | ATCC17415 | CP027714.1 | C4K25_4084 | AZD17004.1 | 100 | 91.51 | C4K25_1943 | AZD28670.1 | 99 | 94.37 |
| *Ps. chlororaphis* | PCL1601 | GCF_001921865.1 | PCL1601_05817 | WP_075120806.1 | 100 | 90.42 | PCL1601_00155 | WP_075118824.1 | 99 | 95.33 |
| *Ps. chlororaphis* | 189 | CP014867.1 | A3218_22750 | AMS16982.1 | 100 | 91.76 | A3218_02970 | AMS13316.1 | 99 | 94.44 |
| *Ps. chlororaphis* | ATCC13985 | LT629738.1 | SAMN04489803_1236 | SDS36014.1 | 100 | 91.76 | SAMN04489803_3667 | SDT28774.1 | 99 | 94.50 |
| *Ps. chlororaphis* | LMG21630 | LT629747.1 | SAMN04489802_2008 | SDS68319.1 | 100 | 91.89 | SAMN04489802_4550 | SDT44975.1 | 99 | 94.50 |
| *Ps. chlororaphis* | PA23 | CP008696.1 | EY04_20910 | AIC21279.1 | 100 | 91.70 | EY04_09280 | AIC19087.1 | 99 | 94.57 |
| **subspecies of *Pseudomonas chlororaphis*** | | |  |  |  |  |  |  |  |  |
| *Pc. aurantiaca* | CW2 | CP027743.1 | C4K20_4305 | AZD49711.1 | 100 | 92.15 | C4K20_1986 | AZD47411.1 | 99 | 94.44 |
| *Pc. aurantiaca* | DSM19603 | CP027746.1 | C4K17_4521 | AZD68398.1 | 100 | 91,89 | C4K17_1955 | AZD65851.1 | 99 | 94.50 |
| *Pc. aurantiaca* | 464 | CP027742.1 | C4K21_4405 | AZD43470.1 | 100 | 91.76 | C4K21_2046 | AZD41130.1 | 99 | 94.76 |
| *Pc. aurantiaca* | JD37 | CP009290.1 | JM49_10315 | AIS12060.1 | 100 | 91.57 | JM49_20570 | AIS13955.1 | 99 | 94.63 |
| *Pc. aurantiaca* | StFRB508 | AP014623.1 | PCAU_4117 | BAV76326.1 | 100 | 92.15 | PCAU_1764 | BAV73973.1 | 99 | 94.57 |
| *Pc. piscium* | ChPhzS135 | CP027738.1 | C4K31_4271 | AZC77165.1 | 100 | 92.08 | C4K31_1968 | AZC74881.1 | 99 | 94.18 |
| *Pc. piscium* | SLPH10 | CP027710.1 | C4K35_4459 | AZC52033.1 | 100 | 92.08 | C4K35_2023 | AZC49616.1 | 99 | 94.18 |
| *Pc. piscium* | PCL1607 | CP027737.1 | C4K32_4286 | AZC70939.1 | 100 | 92.08 | C4K32_2021 | AZC68693.1 | 99 | 94.18 |
| *Pc. piscium* | PCL1391 | CP027736.1 | C4K33_4216 | AZC64699.1 | 100 | 92.08 | C4K33_1954 | AZC62456.1 | 99 | 94.05 |
| *Pc. piscium* | ToZa7 | CP027739.1 | C4K30_4273 | AZC83378.1 | 100 | 92.08 | C4K30_1975 | AZC81099.1 | 99 | 94.05 |
| *Pc. piscium* | ZJU60 | CP027656.1 | C6Q18_20390 | AVO60214.1 | 100 | 92.02 | C6Q18_09505 | AVO58196.1 | 99 | 93.99 |
| *Pc. aureofaciens* | ChPhzS23 | CP027748.1 | C4K09_4044 | AZE18496.1 | 100 | 91.83 | C4K09_1905 | AZE16376.1 | 99 | 94.50 |
| *Pc. aureofaciens* | ChPhzTR18 | CP027751.1 | C4K06_6399 | Non functional | 100 | 91.83 | C4K06_1975 | AZE35018.1 | 99 | 94.57 |
| *Pc. aureofaciens* | ChPhzTR36 | CP027721.1 | C4K12_4357 | AZE00215.1 | 100 | 91.63 | C4K12_2032 | AZD97908.1 | 99 | 94.50 |
| *Pc. aureofaciens* | ChPhzTR38 | CP027752.1 | C4K05_6425 | Non functional | 100 | 91.89 | C4K05_2028 | AZE41378.1 | 99 | 94.57 |
| *Pc. aureofaciens* | 66 | CP027747.1 | C4K10_4246 | AZE12517.1 | 100 | 91.57 | C4K10_2015 | AZE10305.1 | 99 | 94.63 |
| *Pc. aureofaciens* | P2 | CP027719.1 | C4K14_4646 | AZD87461.1 | 100 | 91.70 | C4K14_2030 | AZD84864.1 | 99 | 94.50 |
| *Pc. aureofaciens* | DSM6698 | CP027720.1 | C4K13_4499 | AZD93907.1 | 100 | 91.76 | C4K13_2027 | AZD91454.1 | 99 | 94.50 |

1. In gray, data obtained from under study strain, PCL1606, and its two loci PCL1606_20530 and PCL1606_41090 with which all the loci used in this table have been compared
