# Supplementary material for: Aer Receptors Influence the Pseudomonas chlororaphis PCL1606 Lifestyle
Source: Front Microbiol. 2020 Jul 8;11:1560. doi: 10.3389/fmicb.2020.01560 (PMC7367214; doi:10.3389/fmicb.2020.01560)
Supplement: Supplementary file 5 [file Table_3.DOCX]

TableS3. Swimming motility in soft M9 agar supplemented with 2 mM of carbon source. Motility front diameter values in millimetres, of strains derived from *Pseudomonas chlororaphis* PCL1606 (PcPCL1606) and normalized to the wildtype, considering wildtype as value one.

| **Carbon source** | **PCL1606::aer1-1** | **PCL1606Δaer1-1** | **COM-aer1-1** | **PCL1606::aer1-2** | **PCL1606Δaer1-2** | **COM-aer1-2** | **PCL1606Δaer** |
| --- | --- | --- | --- | --- | --- | --- | --- |
| Arabinose | 1.06±0.04 | 1.08±0.06 | 0.57±0.03* | 0.98±0.07 | 0.96±0.05 | 0.57±0.06* | 1.06±0.07 |
| Fructose | 1.03±0.04 | 1.04±0.04 | 0.76±0.12* | 0.98±0.07 | 0.99±0.09 | 0.84±0.11* | 1.05±0.08 |
| Galactose | 1.03±0.05 | 1.06±0.04 | 0.6±0.06* | 0.97±0.04 | 1±0.03 | 0.68±0.1* | 1.03±0.03 |
| Glycerol | 0.84±0.03* | 0.82±0.06* | 0.99±0.06 | 1±0.06 | 0.95±0.14 | 0.96±0.15 | 0.81±0.04* |
| Glucose | 0.85±0.06* | 0.84±0.05* | 1±0.09 | 0.98±0.07 | 1.04±0.07 | 1.02±0.06 | 0.81±0.05* |
| Sucrose | 1.05±0.06 | 1.05±0.07 | 0.61±0.08* | 0.97±0.06 | 0.98±0.04 | 0.6±0.03* | 1.12±0.05 |
| Xylose | 1.06±0.07 | 1.05±0.06 | 0.53±0.03* | 0.96±0.04 | 0.94±0.06 | 0.51±0.06* | 1.04±0.06 |
| Glutamic acid | 0.86±0.05* | 0.8±0.12* | 0.98±0.09 | 1.02±0.07 | 1.01±0.21 | 0.87±0.11* | 0.8±0.14* |
| Malic acid | 1.02±0.04 | 1.04±0.06 | 0.55±0.05* | 0.96±0.03 | 0.96±0.05 | 0.66±0.08* | 1.03±0.07 |
| Succinic acid | 0.76±0.07* | 0.78±0.12* | 0.92±0.14 | 1.02±0.07 | 1.01±0.06 | 0.99±0.05 | 0.89±0.07* |

*Gray shadow denotes statistically significant differences with PcPCL1606
