# Supplementary material for: Aer Receptors Influence the Pseudomonas chlororaphis PCL1606 Lifestyle
Source: Front Microbiol. 2020 Jul 8;11:1560. doi: 10.3389/fmicb.2020.01560 (PMC7367214; doi:10.3389/fmicb.2020.01560)
Supplement: Supplementary file 6 [file Table_4.DOCX]

TableS4. Swimming motility in soft M9 agar supplemented with 2 mM of carbon source. Motility front diameter values in millimetres, of strains derived from *Pseudomonas chlororaphis* PCL1606 (PcPCL1606) and normalized to the wildtype, considering wildtype as value one.

| **Carbon source** | **1606pBBR** | **1606pLac4** | **1606pLac2** | **PLC1606::aer1-1pBBR** | **PCL1606::aer1-2pBBR** |
| --- | --- | --- | --- | --- | --- |
| Arabinose | 1.01±0.03 | 0.48±0.02* | 0.59±0.05* | 1±0.02 | 1±0.03 |
| Fructose | 1.05±0.05 | 0.47±0.04* | 0.74±0.07* | 0.99±0.08 | 0.97±0.08 |
| Galactose | 1.02±0.03 | 0.55±0.06* | 0.78±0.14* | 0.97±0.07 | 0.97±0.05 |
| Glycerol | 1.06±0.09 | 0.68±0.09* | 0.83±0.11* | 0.85±0.05* | 0.96±0.09 |
| Glucose | 0.99±0.04 | 0.6±0.12* | 0.77±0.05* | 0.76±0.02* | 0.92±0.08 |
| Sucrose | 1.02±0.03 | 0.49±0.07* | 0.64±0.07* | 0.99±0.05 | 0.97±0.05 |
| Xylose | 1±0 | 0.47±0.04* | 0.55±0.05* | 1.01±0.04 | 0.96±0.06 |
| Glutamic acid | 1.08±0.09 | 0.69±0.2* | 0.8±0.09* | 0.8±0.1* | 0.94±0.07 |
| Malic acid | 1±0.03 | 0.52±0.09* | 0.6±0.06* | 1±0.08 | 0.98±0.04 |
| Succinic acid | 1±0.04 | 0.69±0.16* | 0.78±0.12* | 0.87±0.1* | 0.94±0.07 |

*Gray shadow denotes statistically significant differences with PcPCL1606
