# Supplementary material for: Aer Receptors Influence the Pseudomonas chlororaphis PCL1606 Lifestyle
Source: Front Microbiol. 2020 Jul 8;11:1560. doi: 10.3389/fmicb.2020.01560 (PMC7367214; doi:10.3389/fmicb.2020.01560)
Supplement: Supplementary file 7 [file Table_5.DOCX]

Table S5. Results of crystal violet staining along adhesion assay. Average and standard deviation of absorbance at 590nm obtained from bacteria attached on glass surface along 28 h. Further, average and standard deviation of bacterial logarithms at the same time from *Pseudomonas chlororaphis* PCL1606 and derivatives deletional mutants.

|  | **PCL1606** | | **PCL1606Δaer1-1** | | **PCL1606Δaer1-2** | | **PCL1606Δaer** | |
| --- | --- | --- | --- | --- | --- | --- | --- | --- |
| **Hours** | **Abs** | **Log cfu/ml** | **Abs** | **Log cfu/ml** | **Abs** | **Log cfu/ml** | **Abs** | **Log cfu/ml** |
| 0 | 0±0 | 7.37±0.07 | 0±0 | 7.46±0.1 | 0±0 | 7.42±0.03 | 0±0 | 7.43±0.03 |
| 1 | 0.033±0.014 | 7.46±0.08 | 0.021±0.013 | 7.50±0.09 | 0.028±0.016 | 7.47±0.02 | 0.017±0.016 | 7.47±0.04 |
| 2 | 0.044±0.011 | 7.50±0.00 | 0.034±0.009 | 7.62±0.03 | 0.043±0.015 | 7.56±0.01 | 0.021±0.015 | 7.59±0.02 |
| 3 | 0.136±0.019 | 7.49±0.01 | 0.092±0.017 | 7.64±0.05 | 0.106±0.016 | 7.50±0.07 | 0.059±0.022 | 7.60±0.07 |
| 4 | 0.311±0.076 | 7.53±0.10 | 0.245±0.045 | 7.58±0.04 | 0.242±0.022 | 7.54±0.05 | 0.154±0.020 | 7.59±0.02 |
| 5 | 0.353±0.076 | 7.55±0.09 | 0.305±0.038 | 7.53±0.07 | 0.308±0.075 | 7.59±0.07 | 0.184±0.015 | 7.59±0.05 |
| 6 | 0.385±0.063 | 7.58±0.01 | 0.363±0.059 | 7.63±0.00 | 0.386±0.032 | 7.54±0.06 | 0.216±0.036 | 7.61±0.03 |
| 7 | 0.486±0.077 | 7.60±0.06 | 0.425±0.043 | 7.69±0.17 | 0.407±0.061 | 7.65±0.00 | 0.299±0.049 | 7.78±0.11 |
| 8 | 0.589±0.097 | 7.80±0.20 | 0.517±0.065 | 7.84±0.23 | 0.561±0.022 | 7.71±0.13 | 0.388±0.075 | 7.53±0.05 |
| 24 | 0.798±0.184 | 9.14±0.34 | 0.905±0.240 | 8.83±0.16 | 0.788±0.110 | 8.89±0.21 | 0.598±0.069 | 8.81±0.18 |
| 28 | 1.068±0.202 | 9.28±0.20 | 1.232±0.230 | 8.91±0.08 | 1.399±0.120 | 9.11±0.18 | 0.959±0.177 | 9.06±0.22 |
